# *FN1* mRNA 3'-UTR supersedes traditional fibronectin 1 in facilitating the invasion and metastasis of gastric cancer through the *FN1* 3'-UTR-let-7i-5p-THBS1 axis

**DOI:** 10.7150/thno.82492

**Published:** 2023-09-25

**Authors:** Siwei Pan, Jiaming Zhu, Pengfei Liu, Qiaochu Wei, Siyu Zhang, Wen An, Yuxin Tong, Zhenguo Cheng, Funan Liu

**Affiliations:** 1 Department of Surgical Oncology and General Surgery, The First Hospital of China Medical University; Key Laboratory of Precision Diagnosis and Treatment of Gastrointestinal Tumors, China Medical University, Ministry of Education, Shenyang, 110016 China.; 2 Phase I Clinical Trails Center, The First Hospital, China Medical University, 518 North Chuangxin Road, Baita Street, Hunnan District, Shenyang, 110102 Liaoning, China.; 3 Medical Research Center, Liaoning Key Laboratory of Research and Application of Animal Models for Environmental and Metabolic Diseases, Shengjing Hospital of China Medical University, #36 Sanhao Street, Heping District, Shenyang 110004, China.; 4 National Center for International Research in Cell and Gene Therapy, Sino-British Research Centre for Molecular Oncology, State Key Laboratory of Esophageal Cancer Prevention Treatment, School of Basic Medical Sciences, Academy of Medical Sciences, Zhengzhou University, Zhenzhou 450000, China.

**Keywords:** gastric cancer, FN1 3'-UTR, drug target, metastasis, small-molecule drugs

## Abstract

**Background:** Current clinical treatments for gastric cancer (GC), particularly advanced GC, lack infallible therapeutic targets. The 3′-untranslated region (3′-UTR) has attracted increasing attention as a drug target.

**Methods:** *In vitro* and *in vivo* experiments were conducted to determine the function of *FN1* 3′**-**UTR and FN1 protein in invasion and metastasis. RNA pull-down assay and high-throughput sequencing were used to screen the factors regulated by *FN1* 3′**-**UTR and construct the regulatory network. Western blotting and polymerase chain reaction were used to examine the correlation of intermolecular expression levels. RNA-binding protein immunoprecipitation was used to verify the correlation between *FN1* 3′**-**UTR and target mRNAs.

**Results:** The *FN1* 3′-UTR may have stronger prognostic implications than the FN1 protein in GC patients. Upregulation of *FN1* 3′**-**UTR significantly promoted the invasive and metastatic abilities of GC cells to a greater extent than FN1 protein *in vitro* and *in vivo*. A novel regulatory network was constructed based on the *FN1* 3′**-**UTR-let-7i-5p-THBS1 axis, wherein *FN1* 3′**-**UTR displayed stronger oncogenic effects than the FN1 protein.

**Conclusions:** *FN1* 3′-UTR may be a better therapeutic target for constructing targeted drugs in GC than the FN1 protein.

## Introduction

There were over one million new gastric cancer (GC) cases and 769,000 deaths from the disease worldwide in 2020. This ranks fifth among cancer-associated morbidities and is the fourth leading cause of mortality among all malignant tumors. The five-year overall survival (OS) of stage IV disease is dismal 1-3. Most first-line treatments for unresectable GC are based on the combination of fluorouracil and platinum. Moreover, the application of second- or third-line regimens may fall outside the most effective window for treatment in the event of partial progression or chemotherapy resistance 4. Traditional therapies for GC often target proteins or their related pathways. These include apatinib (a selective VEGFR2 tyrosine kinase inhibitor (TKI) for advanced GC) 5, regorafenib (a multikinase inhibitor of angiogenic and oncogenic kinases including FGFR) 6, and sorafenib (a well-known tyrosine kinase-inhibiting anti-cancer drug targeting the Ras/Raf/Mek/Erk cascade pathway) 7. However, these traditional therapies have low efficacy, mainly due to their antagonistic protein-targeting effects. Therefore, targeting deeper transcriptomes rather than proteins may have promising therapeutic implications.

Protein encoding mRNAs and their 3′-untranslated region (3′-UTR) domains play distinct roles in tumor development 8-10. Moreover, mRNA processing is altered in cancer, mainly by interfering with the 3′-UTR expression of genes. The resulting pro- or anti-cancer effects are mediated via mutation of mRNA splicing factor genes or shortening of 3′**-**UTRs 11, 12. Studies have revealed that 3′-UTR regions harbor binding sites for microRNAs (miRNAs), such as miR-132-3p, miR-200c-3p, and miR-1-3p 13-16. Prediction of miRNA binding on the 3′-UTR region using the TargetScan 7.2 database was also employed 17. Targeting 3′-UTR can induce tumor cells to become sensitive to chemotherapeutic drugs 18. Some immunomodulatory drugs were proposed for treating diseases by targeting the 3′-UTR 19, 20. Inhibitors targeting proteins, such as lapatinib in HER2-positive GC [21] and sunitinib (a multi-targeted TKI) fail to treat metastatic gastrointestinal stromal tumors [22]. Therefore, we hypothesized that drugs targeting the 3′-UTR might be better therapeutic agents for GC treatment.

Fibronectin (FN) is a multifunctional protein located in the extracellular matrix (ECM). It primarily originates from cancer-associated fibroblasts (CAFs) and epithelial cells in the tumor microenvironment (TME) 21, 22. FN1 forms a scaffold structure and binds to cell-surface integrins to induce cell migration 23-25. Our previous study 15 showed no significant correlation between the FN1 protein level and the prognosis of GC patients. We found that high *FN1* mRNA levels significantly negatively affected patient prognosis, which was consistent with a previous study 26.

This study aimed to determine the function of *FN1* RNA and clinical significance of its levels in GC. We found that *FN1* 3′-UTR is a predominant factor that affects the invasion and metastasis of GC. Our results provide evidence that targeting *FN1* 3′-UTR may have greater therapeutic efficacy than targeting the FN1 protein.

## Results

### *FN1* mRNA has a greater clinical relevance than the FN1 protein

**Figure 1:**
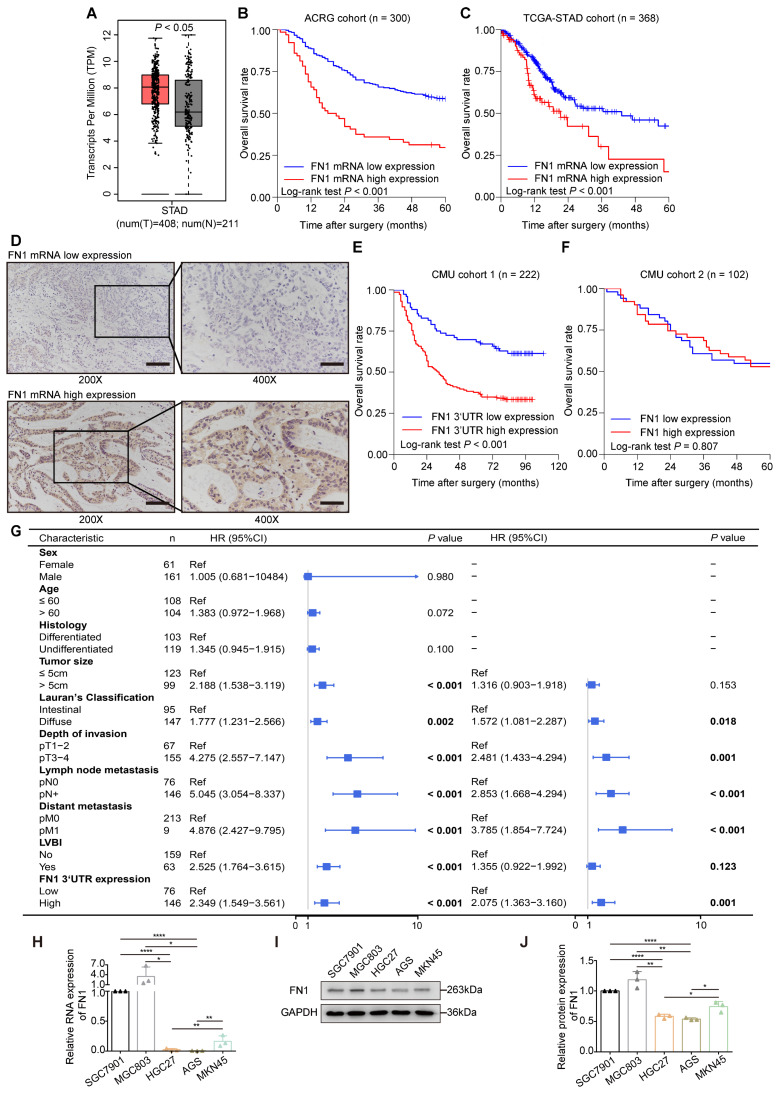
** Expression levels and clinical significance of *FN1* mRNA and protein.** *FN1* mRNA levels compared between GC tissues and adjacent tissues from TCGA-STAD and GTEx databases (A). Kaplan-Meier curves of low and high *FN1* mRNA expression in the ACRG (B) and TCGA-STAD (C) cohorts. *In situ* hybridization assays for the different expression levels of *FN1* mRNA in GC tissues of CMU cohort1 (n = 222). 200X: scale bars = 100μm; 400X: scale bars = 50μm (D). Kaplan-Meier curves of low and high expression levels of *FN1* 3'-UTR in CMU cohort1 (E) and FN1 protein in CMU cohort2 (F). Univariate and multivariate analyses of the Cox proportional hazards model and forest plot of hazard ratios (HRs) and 95% confidence intervals for the overall survival of the CMU cohort1 (G). The relative expression levels of *FN1* mRNA, determined by real-time PCR in GC cell lines (H). The relative protein levels of FN1 in GC cell lines, determined by western blotting and analyzed using ImageJ software (I, J). The data are presented as a histogram of the mean ± SEM of three independent experiments in H and J and compared using Student's *t*-test (*P < 0.05, ** P < 0.01, *** P < 0.001, **** P < 0.0001, n = 3).

The application of the Gene Expression Profile Interactive Analysis (GEPIA) platform 27 and clinical patient tissue validation revealed that *FN1* mRNA expression was significantly higher in cancer tissues than in adjacent tissues (**Figure 1A**). We speculated that *FN1* mRNA might play a role in promoting tumors in GC cells, regardless of whether it is mutated. The correlation between *FN1* mRNA levels and the prognosis of GC patients was performed using bioinformatic analyses of the Asian Cancer Research Group (ACRG) and TCGA-STAD cohorts. Survival analysis showed that postoperative survival was significantly better in patients with low *FN1* mRNA expression than in patients with high expression in the ACRG (five-year OS: 58.4% vs. 29.6%, *P* < 0.001; **Figure 1B**) and TCGA-STAD (five-year OS: 42.5% vs. 15.1%, *P* < 0.001; **Figure 1C**) cohorts. *FN1* mRNA expression was evaluated by targeting the *FN1* 3'-UTR and coding sequence (CDS) regions respectively, in 222 GC tissues via *in situ* hybridization (ISH) [Chinese Medical University (CMU) cohort 1] (**Figure 1D**). The patients (65.8%) that exhibited high *FN1* 3'-UTR expression were more likely to have provocative invasion capacity (pT stage, *P* = 0.025) and metastatic lymph nodes (pN stage, *P* = 0.031) (**Table S1**). Low *FN1* 3′-UTR expression consistently and significantly correlated with high OS (five-year OS: 68.4% vs. 37.7%, *P* < 0.001, **Figure 1E**). However, there was no significant difference in survival between patients with high or low expression of *FN1* CDS (*P* = 0.118, **Figure S1**). Correlation analysis of the clinicopathological characteristics in the *FN1* CDS region was comparable with the pT-stage of GC patients (*P* = 0.042, **Table S1**); this was consistent with the results of FN1 protein. Analysis of FN1 protein expression in 102 GC patients (CMU cohort 2) from our published data 15 showed that FN1 protein levels only correlated with the pT stage (*P* = 0.028, **Table S1**). No significant prognostic differences were noted in patients expressing different levels of FN1 protein (five-year OS: 54.9% vs. 52.9%, *P* = 0.807, **Figure 1F**). Moreover, Cox multivariate analysis revealed that elevated *FN1* mRNA expression was an independent predictor of poor prognosis in GC [HR (hazard ratio [HR]) = 2.075, 95% confidence interval (CI) = 1.363-3.160, *P* = 0.001; **Figure 1G**]. The expression of *FN1* 3′**-**UTRs was initially analyzed within a set of GC cell lines to explore their biological functions compared with the FN1 protein (**Figure 1H-1J**). HGC27 and AGS cells were selected for *in vitro* functional assays, as they exhibit the lowest level of *FN1* 3′**-**UTR and FN1 expression. Notably, the relative rates at the *FN1* mRNA level significantly differed from the FN1 protein level across cell lines. We speculated that this might be related to the high mutation rate of *FN1* mRNA in GC.

### Contradistinction of *FN1* mRNA and FN1 protein biological function* in vitro* and* in vivo*

**Figure 2:**
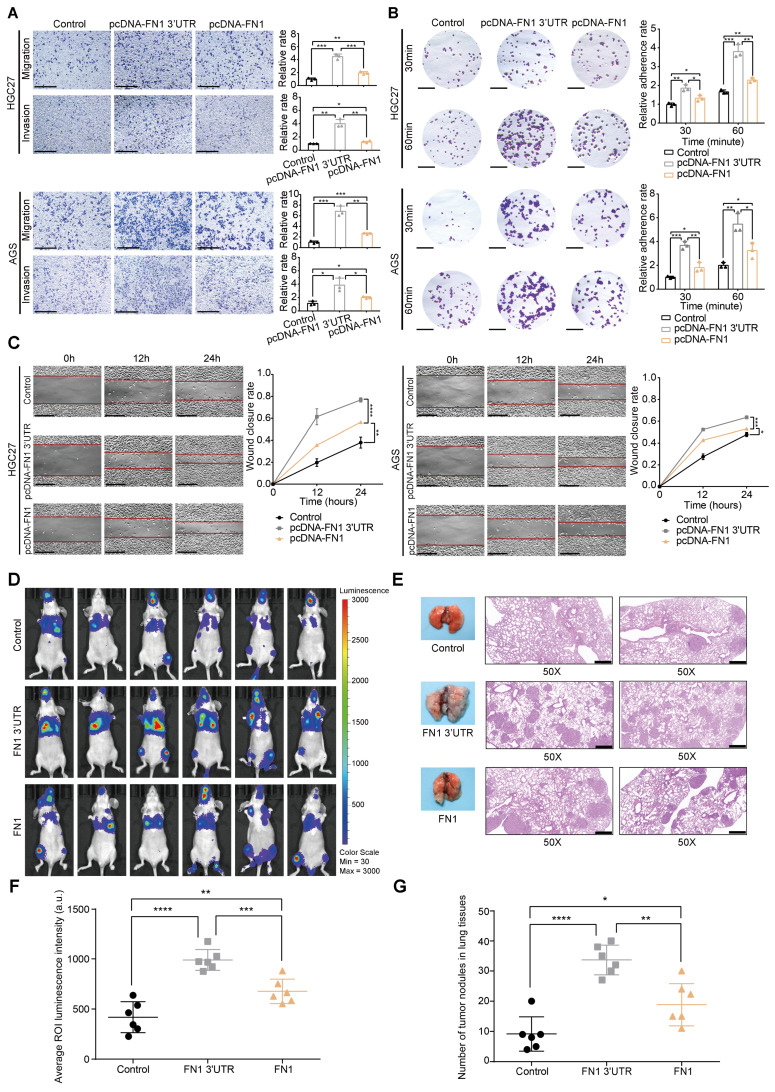
***FN1* 3'-UTR may play a more relevant clinical role in promoting GC than FN1 protein.** Comparison of the migration and invasion abilities between the *FN1* 3′-UTR overexpressed group, the FN1 protein overexpressed group, and the negative control group of HGC27 and AGS cells analyzed using ImageJ software. Scale bars = 500μm (A). The adhesion ability (scale bars = 1000μm) (B) and surface mobilities (scale bars = 500μm) (C) of the *FN1* 3′-UTR overexpressed group, FN1 protein overexpressed group, and negative control group analyzed using ImageJ software. Tumor volume was monitored using the *in vivo* optical imaging system for 9 weeks after tail vein injection (D). The gross lesions of lung tissues isolated from mice and microscopic images of lung tissue sections stained with hematoxylin and eosin. Scale bars = 500μm (E). The average ROI (regions of interest) luminescence intensity obtained by *in vivo* imaging of the *FN1* 3′-UTR overexpressed group, the FN1 protein overexpressed group, and the negative control group (F). The number of metastatic nodules in the lungs (G). The data are presented as a histogram of the mean ± SEM of three independent experiments in A-C and compared using Student's *t*-test (*P < 0.05, ** P < 0.01, *** P < 0.001, **** P < 0.0001, n = 3).

HGC27 and AGS cells were selected for further *in vitro* functional assays by transfecting plasmids overexpressing the *FN1* CDS and 3′-UTR regions, respectively (**Figure S2A-S2D**). Although an equal amount of plasmids were transfected into both cell lines, *FN1* CDS showed much higher expression than that of *FN1* 3′**-**UTR at the transcriptional level (**Figure S2B**). Cells overexpressing *FN1* 3′-UTR displayed more aggressive migration and invasion than those expressing FN1 (**Figure 2A**). Similarly, the adhesion ability and surface mobility of cells overexpressing *FN1* 3′-UTR was much higher than cells overexpressing FN1 protein (**Figure 2B-2C**). *In vivo* xenograft assays showed that nude mice subjected to tail vein injection of HGC27 cells stably overexpressing *FN1* 3′-UTR displayed greater numbers of lung metastatic and peripheral microcirculation colonies than those injected with HGC27 cells stably overexpressing FN1 protein (n = 6 for each group) (**Figure 2D-2G**). These results demonstrate that* FN1* 3′-UTR plays a more significant role in metastasis than *FN1* CDS.

### *FN1* 3′-UTR overexpression affects cellular and signaling pathways related to invasion and metastasis

The HGC27 cell line stably overexpressing *FN1* 3′-UTR was used for high-throughput RNA-seq to discern the specific molecular mechanisms underlying the effect of *FN1* 3′-UTR in facilitating invasion and metastasis (**Table S2**). *FN1* 3′-UTR overexpression led to 323 significantly differentially expressed genes (DEGs: 241 genes were upregulated and 82 genes were downregulated) based on the filtering criteria of |logFoldChange| > 1 and *P* < 0.05, (**Table S3, Figure S3A-S3B**). Gene Ontology (GO) enrichment and Kyoto Encyclopedia of Genes and Genomes (KEGG) pathway analyses of the 323 DEGs showed that they are involved in several cellular functions and signaling pathways related to invasion and metastasis, including ECM organization, cell adhesion molecule binding, ECM receptor interaction, and the transforming growth factor β (TGFβ) signaling pathway (**Figure S3C-S3D**). Additionally, Gene Set Enrichment Analysis (GSEA) of RNA-seq data revealed that *FN1* 3′-UTR overexpression may affect epithelial-mesenchymal transition (EMT)-related signaling pathways, the TGFβ signaling pathway, receptor interaction in the ECM, and protein secretion (**Figure S3E**). These results led us to hypothesize that *FN1* 3'-UTR may be the main component of FN1, which was previously shown to play a role in promoting EMT, TGFβ expression, and invasion.

### *FN1* 3′-UTR induces GC cell-mediated changes in peritoneal integrity

**Figure 3:**
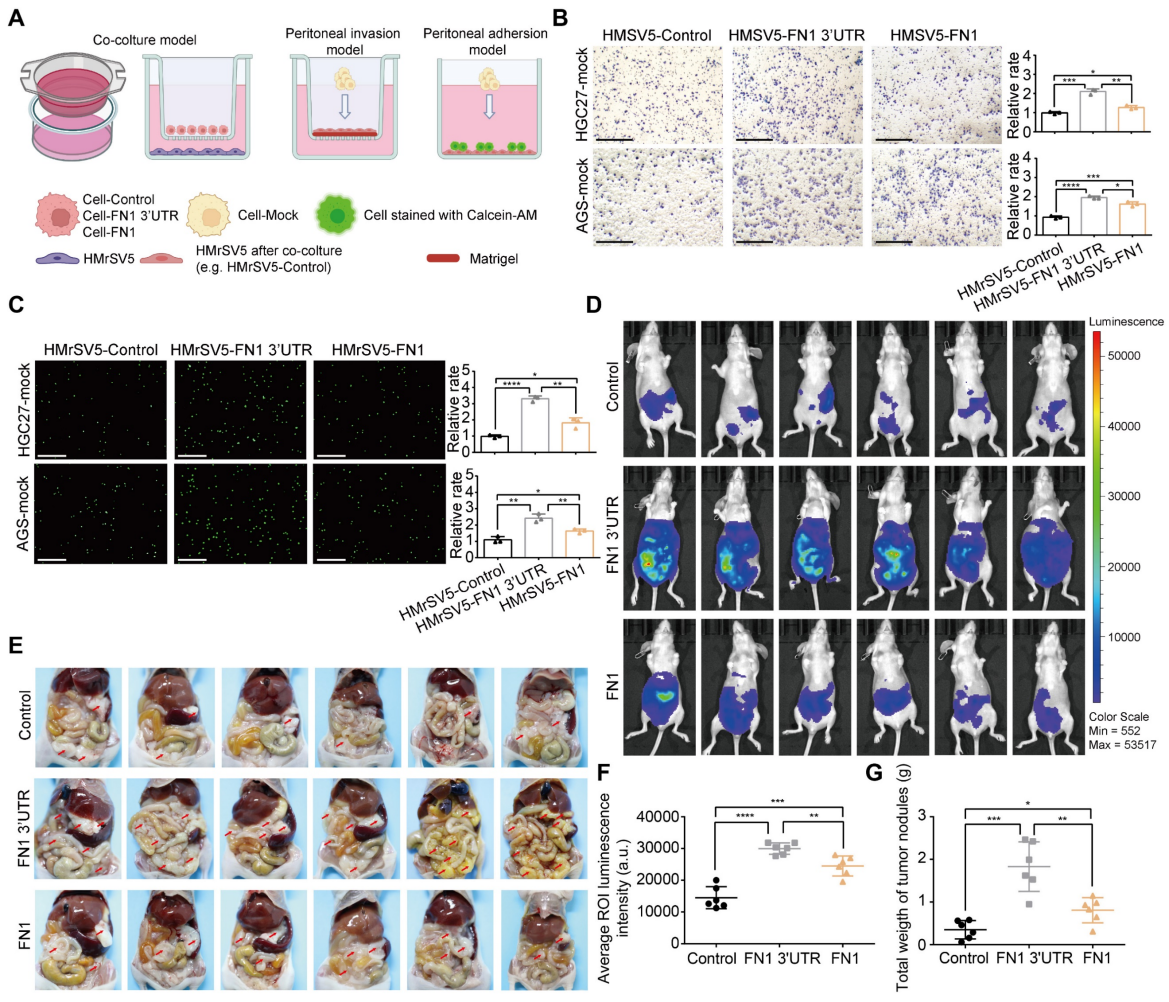
***FN1* 3'-UTR in GC cells may affect mesothelial cells in the peritoneal environment.** Experimental model of cocultured GC cells and mesothelial cells (A). The ability of HGC27 and AGS cells adhering to HPMCs (scale bars = 500μm) (B) and invading HPMCs (scale bars = 1000μm) (C), which were cocultured with different GC cells and analyzed using ImageJ software. Tumor volume was monitored using the *in vivo* optical imaging system for 5 weeks after intraperitoneal injection (D) and representative tumor nodules were marked in the abdominal cavity of nude mice in different groups (E). The average ROI luminescence intensity obtained by *in vivo* imaging in the *FN1* 3′-UTR overexpressed group (F). Differences of the tumor nodule weights between the *FN1* 3′-UTR overexpressed group, the FN1 protein overexpressed group, and the negative control group (G). The data are presented as a histogram of the mean ± SEM of three independent experiments in B and C and compared using Student's *t*-test (*P < 0.05, ** P < 0.01, *** P < 0.001, **** P < 0.0001, n = 3).

GC cells commonly detach from the external wall of the stomach and are implanted in the peritoneum; this leads to peritoneal metastasis and a very poor prognosis 28. A layer of mesothelial cells on the peritoneal surface serves as a physical barrier. However, these cells are prone to a series of changes such as senescence, autophagy, and EMT under the long-term effects of the TME, including higher amounts of TGFβ secreted by GC cells, which makes it easier for GC cells to invade the peritoneum and become implanted 29-31. Therefore, HMrSV5 cells were cocultured with HGC27 or AGS cells under the indicated conditions to investigate the GC cell-mediated alterations in invasion resistance and the adhesion ability of peritoneal mesothelial cells (**Figure 3A**). The results obtained from the peritoneal invasion model revealed that under *FN1* 3′-UTR stimulation, HMrSV5 cells showed notably weaker resistance to invasion by HGC27 or AGS cells than under overexpressed FN1 protein, indicating that the HMrSV5-FN1 3′-UTR group had the weakest barrier ability (**Figure 3B**). The peritoneal adhesion model showed that HMrSV5-FN1 3′-UTR possessed a stronger ability to adhere with HGC27 or AGS cells than the HMrSV5-FN1 group (**Figure 3C**). *In vivo* assays also demonstrated that *FN1* 3′-UTR overexpression increased the number of macroscopic nodules and resulted in a significantly higher tumor weight during peritoneal cavity dissemination to the mesenterium, greater omentum, and parietal peritoneum compared with the FN1 protein overexpression group and negative control group (n = 6 for each group) (**Figure 3D-3G**).

### *FN1* 3′-UTR serves as a core network regulator in GC

Competing endogenous RNAs (ceRNAs) form a large-scale regulatory network across the transcriptome and play key roles in various pathologies, diseases, and cancer 32-35. Recently, the importance of mRNA 3′-UTR as a ceRNA in tumor development was emphasized in addition to ceRNA studies targeting long noncoding RNAs (lncRNAs) and circular RNAs (circRNAs) 36-38.

**Figure 4:**
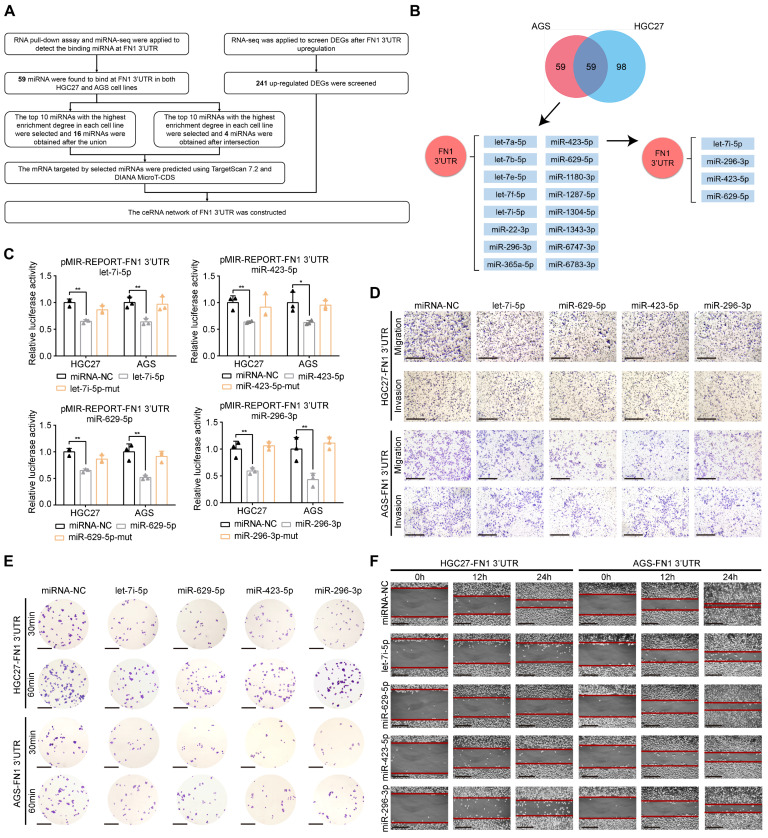
***FN1* 3'-UTR serves as the core regulator of a network and sponges various microRNAs in GC.** Flow chart showing the analyses of miRNA-seq and RNA-seq data (A). Venn plot of the number of enriched miRNAs in HGC27 and AGS cell lines, and the regulatory network among the *FN1* 3′-UTR and various miRNAs (B). Luciferase activity in GC cells co-transfected with the luciferase reporter containing the *FN1* 3′-UTR and the mimics of let-7i-5p, miR-423-5p, miR-629-5p, miR-296-3p, or mutant. Data are presented as the relative ratio of *Renilla* luciferase activity (C). The alterations of the migration and invasion abilities of *FN1* 3′-UTR overexpressed cells after the transfection of four miRNA mimics. Scale bars = 500μm (D). The adhesion ability at 30 min or 60 min (scale bars = 1000μm) (E) and surface mobilities (scale bars = 500μm) (F) of *FN1* 3′-UTR overexpressed cells after transfecting the four miRNA mimics. The data are presented as a histogram of the mean ± SEM of three independent experiments in C and compared using Student's *t*-test (*P < 0.05, ** P < 0.01, *** P < 0.001, **** P < 0.0001, n = 3).

Next, we aimed to detemine whether* FN1* 3'-UTR acts in GC via ceRNA regulation. To this end, miRNA-sequencing (miR-seq) was performed on HGC27/AGS-FN1 3′-UTR cells after enrichment of* FN1* 3′-UTR via an RNA pull-down assay (**Table S4-S5**). Combined analysis of miRNA-seq and RNA-seq data was performed to construct a network with *FN1* 3′-UTR at its core (**Figure 4A**). One hundred and fifty-seven miRNAs and 118 miRNAs were highly enriched in the *FN1* 3′-UTRs of HGC27-FN1 3′-UTR and AGS-FN1 3′-UTR cells, respectively. A total of 59 common miRNAs were selected from both HGC27 and AGS cells. Separate screening of the 10 most enriched miRNAs in both cell lines defined 16 miRNAs in both cell lines, while four miRNAs were obtained at an intersection (**Figure 4B and Table S6**). The authenticity of this proposed regulatory network was verified by initially predicting the binding sites of the four intersecting miRNAs on the *FN1* 3'-UTR region via RNA22 39, a database for detecting miRNA targets (**Figure S4A**). Luciferase reporter assays confirmed the predictions, as luciferase activity was inhibited by the four miRNA-mimics, while no changes were observed in the group transfected with the four miRNA-mimic-muts (**Figure 4C**). The four miRNA mimics were transfected in HGC27 and AGS cells, and their transfection efficiencies were verified. *FN1* 3′-UTR expression was significantly downregulated in cells transfected with the mimics (**Figure S4B-S4C**). Further functional assays in HGC27 and AGS cells demonstrated that the transfection of let-7i-5p mimics eliminated the effect of *FN1* 3′-UTR toward promoting cellular invasiveness, adhesion, and mobility (**Figure 4D-4F and Figure S4D-S4G**). Similar results were obtained in experiments involving three other miRNAs (miR-629-5p, miR-423-5p, and miR-296-3p). Collectively, these results suggest a negative regulatory role for the *FN1* 3′-UTR-miRNA axis in GC cells. This provides a molecular basis for *FN1* 3′-UTR to function via the ceRNA regulatory network.

### *THBS1, CPED1*, and *AMOTL2* expression is regulated by* FN1* 3′-UTR within the ceRNA network

**Figure 5:**
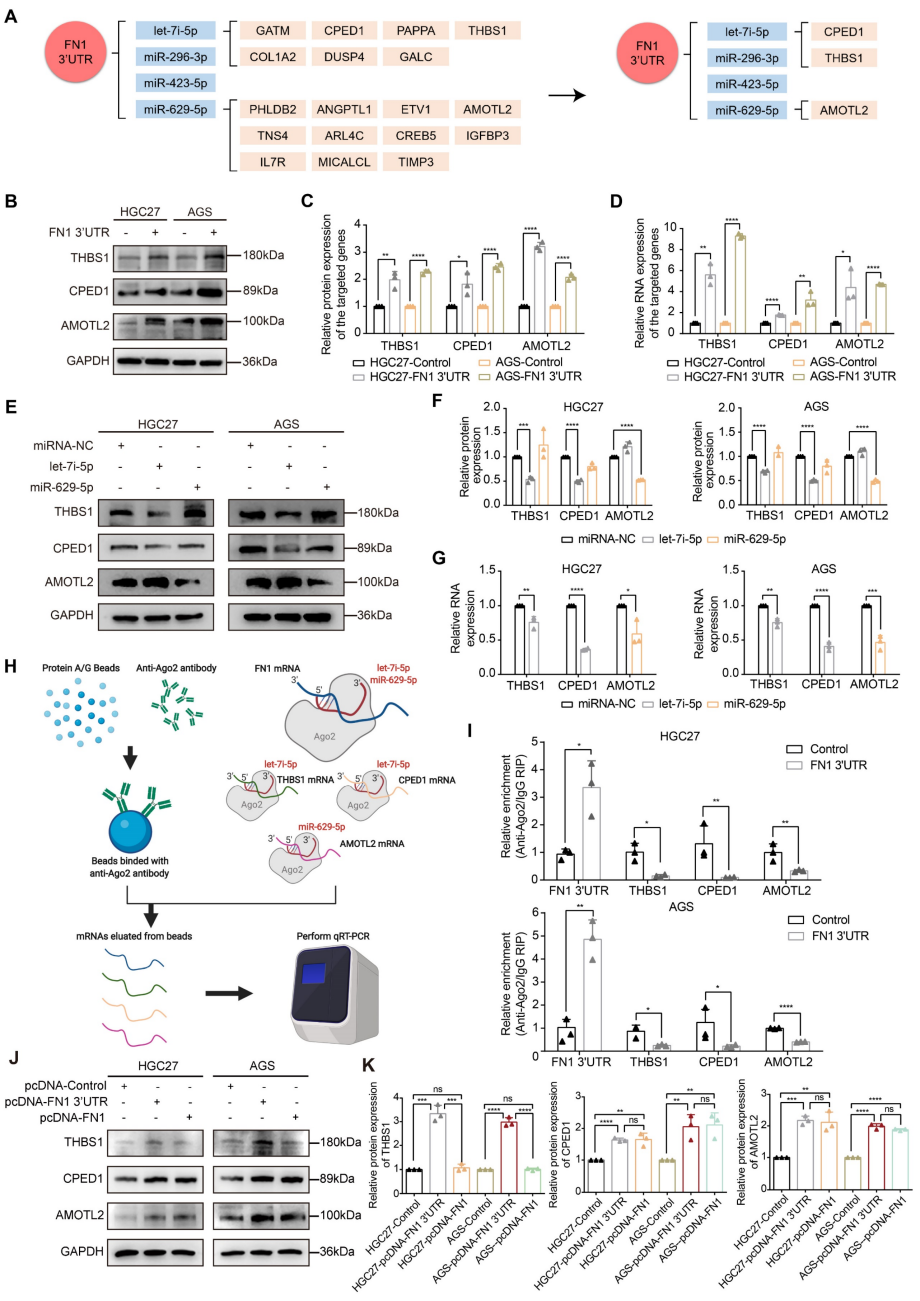
***THBS1*, *CPED1*, and *AMOTL2* were regulated by *FN1* 3'-UTR via let-7i-5p and miR-629-5p.** The oncogenic and core regulatory network using *FN1* 3′-UTR as the core (A). The relative expression levels of THBS1, CPED1, and AMOTL2 determined by western blotting, ImageJ software analysis, and real-time PCR in *FN1* 3′-UTR overexpressed and negative cells (B, C, D). The regulatory relationships between let-7i-5p and THBS1, let-7i-5p and CPED1, and miR-629-5p and AMOTL2 in the core subnetwork were validated by western blot analysis and real-time PCR (E, F, G). Schematic diagram (H) and real-time PCR results (I) of the RNA immunoprecipitation chip assay based on AGO2. The relative expression levels of THBS1, CPED1, and AMOTL2 determined by western blot analysis and ImageJ software in *FN1* 3′-UTR overexpressed cells, FN1 protein overexpressed cells, and negative control cells (J, K). The data are presented as a histogram of the mean ± SEM of three independent experiments in C, D, F, G, I, and K and compared using Student's *t*-test (*P < 0.05, ** P < 0.01, *** P < 0.001, **** P < 0.0001, n = 3).

We used miRNA-seq to analyze HGC27/AGS-FN1 3′-UTR cells and obtained 16 target miRNAs in the above (**Figure 4B and Table S6**). Target genes of these miRNAs were predicted using TargetScan7.2 17 and DIANA MicroT-CDS 40 databases (**Table S7-S22**). There were 66 target genes that overlapped with the 241 upregulated DEGs selected from RNA-seq analysis of the HGC27 cells stably overexpressing *FN1* 3′-UTR (**Table S23**). Thus, a novel ceRNA regulatory network containing 16 miRNAs and 66 target genes was established using *FN1* 3′-UTR as the core (**Figure S5**). Secondly, *FN1* 3′-UTR showed an oncogenic role based on *in vivo* and *in vitro* experiments. This prompted us to select highly expressed target genes that are significantly associated with poor prognosis in GC patients. Screening of these 66 target genes using expression profiles and prognostic information from the ACRG cohort yielded 14 genes (**Table S23**). Consequently, an oncogenic subnetwork was constructed, including 16 miRNAs and 14 regulated genes (**Figure 5A**). Thirdly, screening of the top 10 enriched miRNAs from the miRNA-seq results, separately performed on HGC27-FN1 3′-UTR cells and AGS-FN1 3′-UTR cells, yielded four miRNAs after an intersection (**Figure 4B**). Subsequently, only three target genes were selected from the above 14 target genes: *THBS1*, *CPED1,* and *AMOTL2* (**Figure 5A**). To verify the regulation of the three target genes by the *FN1* 3′-UTR via the ceRNA regulatory subnetwork, the alterations in their expression in HGC27-FN1 3′-UTR and AGS-FN1 3′-UTR cells were examined. The expression of *THBS1*, *CPED1*, and *AMOTL2* was significantly upregulated in cells overexpressing* FN1* 3′-UTR compared with the negative control cells (**Figure 5B-5D**). This was consistent with the RNA-seq results. The ceRNA networks mainly regulate target genes by noncoding RNAs via sponging miRNAs. This weakens the effect of miRNA on target genes. Considering this, the regulatory relationships between miRNAs and target genes in the ceRNA network were validated. *In vitro* experiments using HGC27 and AGS cells confirmed that the let-7i-5p transfection decreased the expression of *THBS1* and *CPED1*, while the miR-629-5p transfection reduced *AMOTL2* levels (**Figure 5E-5G**). Moreover, RNA immunoprecipitation chip (RIP) assays based on AGO2 (which can recruit target mRNA by binding miRNAs upon immunoprecipitation) confirmed a ceRNA involvement between* FN1* 3′-UTR and the three target genes (**Figure 5H**). Furthermore, *FN1* 3′-UTR overexpression elicited a significant decrease in the enrichment of *THBS1*, *CPED1*, and *AMOTL2* mRNAs, which were pulled down by the anti-AGO2 antibody in HGC27 and AGS cells (**Figure 5I**). These results indicated that miRNA-bound targeted genes may be transcriptionally repressed under *FN1* 3′-UTR stimulation. This suggests that *FN1* 3′-UTR competes with *THBS1, CPED1*, and *AMOTL2* mRNAs for miRNA binding and results in upregulation of these three targeted genes; this verified that our novel predicted ceRNA network has practical value. We hypothesized that the differential function and signaling mechanism of FN1 at the RNA and protein level might be related to the ceRNA mechanism of* FN1* 3′-UTR. Our results clarified the regulatory role of *FN1* 3′-UTR on the three targeted genes. *CPED1* and *AMOTL2* were upregulated in cells overexpressing *FN1* 3′-UTR and FN1 protein compared with the negative control cells. However, *THBS1* was not significantly altered in the FN1 protein overexpression group and was significantly lower than that in *FN1* 3′-UTR overexpressing cells (**Figure 5J-5K**).

### The *FN1* 3′-UTR-let-7i-5p-THBS1 axis may be the pivotal mechanism by which the *FN1* 3′-UTR affects GC invasion and metastasis

**Figure 6:**
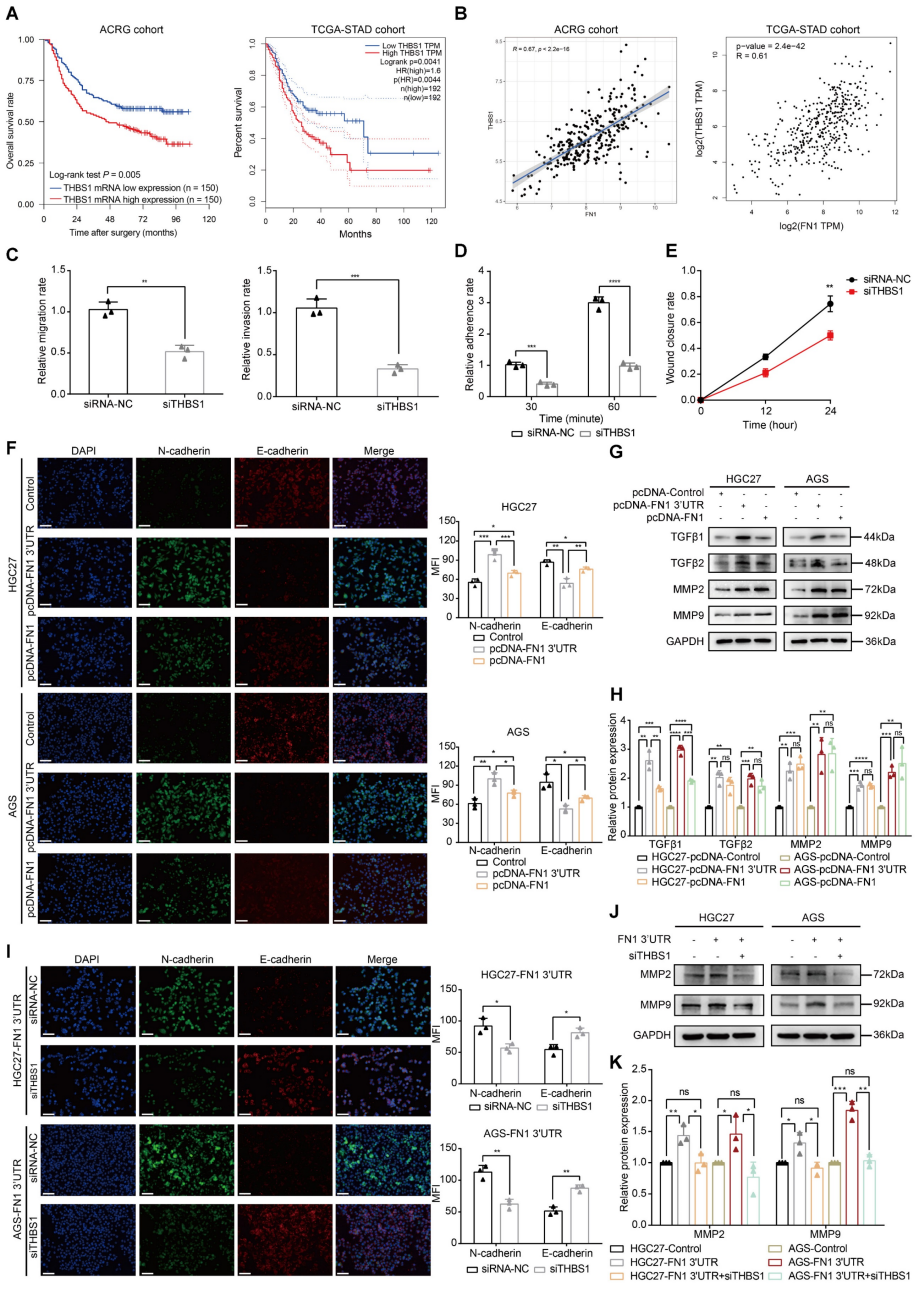
** The *FN1* 3'-UTR-let-7i-5p-THBS1 axis may be the central regulatory network accounting for the differences in the effects of overexpressed FN1 protein.** Kaplan-Meier curves of low and high *THBS1* expression in the ACRG and TCGA-STAD cohorts (A). Correlation between the expression of *THBS1* mRNA and *FN1* mRNA in the ACRG and TCGA-STAD cohorts analyzed using Spearman's rank correlation test (B). The migration and invasion abilities (C), adhesion ability (D), and surface mobilities (E) of *FN1* 3′-UTR overexpressed HGC27 cells after siTHBS1 transfection. Immunofluorescence microscopy of epithelial-mesenchymal transition (EMT) marker expression in *FN1* 3′-UTR overexpressed cells, FN1 protein overexpressed cells, and negative control cells. The mean fluorescence intensities (MFIs) of N-cadherin or E-cadherin measured using ImageJ software and compared using Student's *t*-test. Scale bars = 100μm (F). The relative protein levels of TGFβ1, TGFβ2, MMP2, and MMP9 determined by western blotting and ImageJ software analysis using *FN1* 3′-UTR overexpressed cells, FN1 protein overexpressed cells, and negative control cells (G, H). Immunofluorescence microscopy to determine the differential expression of EMT markers after the transfection of siTHBS1 in *FN1* 3′-UTR overexpressed cells. The mean fluorescence intensities (MFIs) of N-cadherin or E-cadherin measured using ImageJ software and compared using Student's *t*-test. Scale bars = 100μm (I). Differential expression of MMP2 and MMP9 after transfecting *FN1* 3′-UTR overexpressing cells with siTHBS1 determined by western blotting and ImageJ software analysis (J, K). The data are presented as a histogram of the mean ± SEM from three independent experiments in C-K and compared using Student's *t*-test (*P < 0.05, ** P < 0.01, *** P < 0.001, **** P < 0.0001, n = 3).

The molecular mechanism of* FN1* 3′-UTR was clarified by subjecting the three target genes to bioinformatic analysis to explore the core factors of the regulatory network. *THBS1* expression levels were significantly associated with the prognosis of patients with GC: a higher expression was associated with a poorer prognosis in the ACRG (*P* = 0.005) and TCGA-STAD (*P* = 0.0041) cohorts (**Figure 6A**). In contrast, *CPED1* (*P* = 0.012) and *AMOTL2* (*P* = 0.038) were significantly correlated with prognosis only in the ACRG cohort (**Figure S6A**). Correlation analysis of *FN1* mRNA expression showed that all three target genes were significantly correlated with *FN1* mRNA (R > 0 and *P* < 0.05), with *THBS1* showing the highest correlation in both cohorts (ACRG cohort: R = 0.67, TCGA-STAD cohort: R = 0.61, **Figure 6B** and **Figure S6B**). Therefore, we propose that *THBS1* might play a central role in this network and modulate the functional differences between *FN1* 3'-UTR and FN1 protein, as it is high correlated with *FN1* mRNA expression and based on its high clinical significance. *In vitro* experiments performed after downregulating *THBS1* in HGC27/AGS-FN1 3′-UTR cells using small interfering RNA (siRNA) (**Figure S7A-S7C**) indicated that siRNA-*THBS1* partially eliminated the inducing effect of *FN1* 3′-UTR on cellular invasiveness, cellular adhesion, and surface mobility (**Figure 6C-6E and Figure S7D-S7F**).

**Figure 7:**
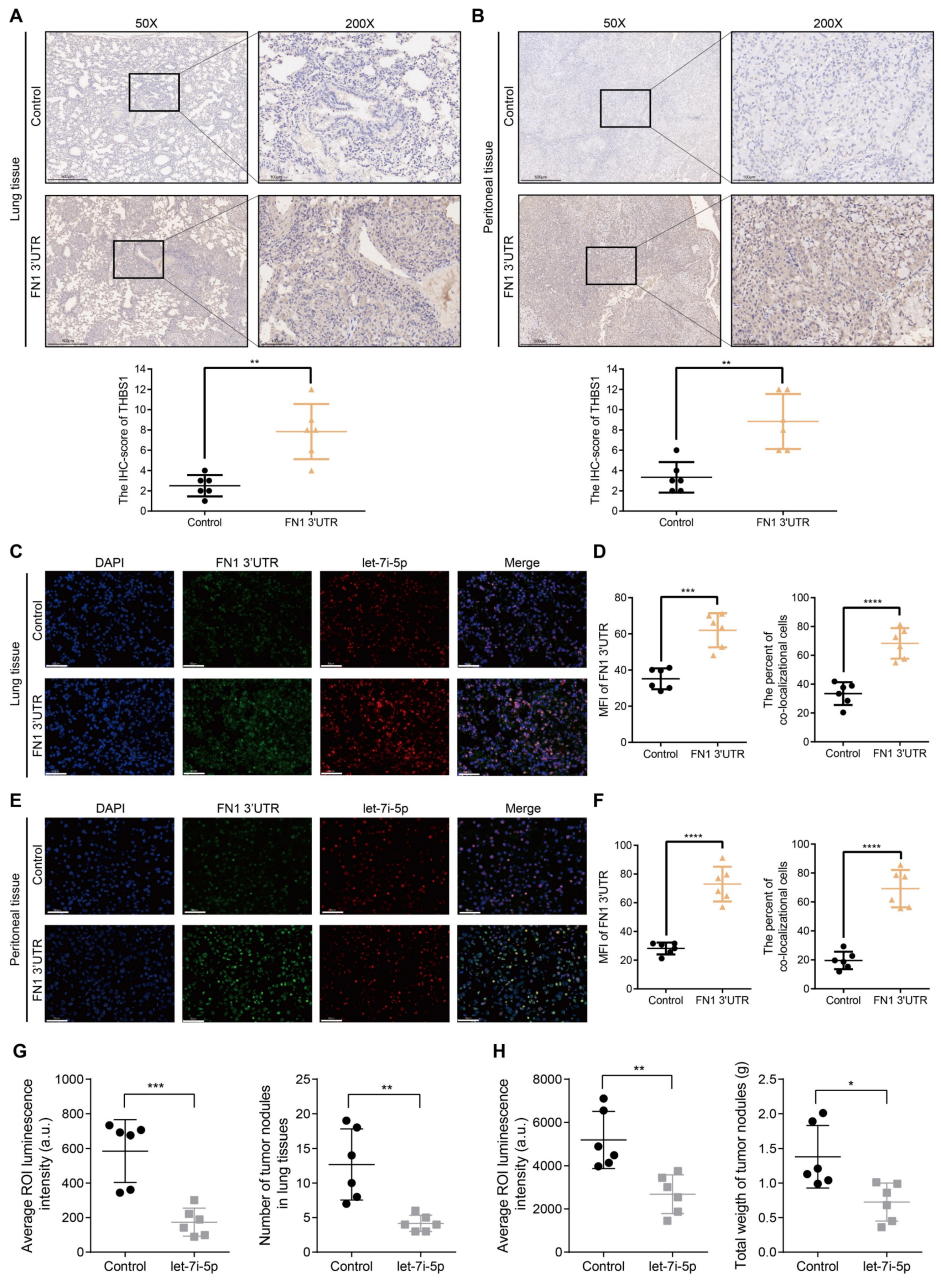
** The *FN1* 3′-UTR-let-7i-5p-THBS1 axis exists *in vivo* and plays a role in promoting tumor metastasis.** Immunohistochemical results of THBS1 expression in the metastatic nodules in lungs (50X: scale bars = 500μm; 200X: scale bars = 100μm) (A) and tumor nodules in the abdominal cavity (50X: scale bars = 500μm; 200X: scale bars = 100μm) (B). Immunofluorescence microscopy and analysis of the co-localization between *FN1* 3'-UTR and let-7i-5p in the metastatic nodules of lungs from the *FN1* 3′-UTR overexpression group and the negative control group. Scale bars = 50μm (C-D). Immunofluorescence microscopy and analysis of the co-localization between *FN1* 3'-UTR and let-7i-5p in the tumor nodules of the abdominal cavity from the *FN1* 3′-UTR overexpression group and the negative control group. Scale bars = 50μm (E-F). The average ROI luminescence intensity obtained by *in vivo* imaging and the number of metastatic nodules in the lungs of let-7i-5p overexpressed group in metastasis assays (G). The average ROI luminescence intensity obtained by *in vivo* imaging and the weights of tumor nodule of let-7i-5p overexpressed group in peritoneal implantation assays (H). The data are presented as a histogram of the mean ± SEM of six independent experiments in A-C and compared using Student's *t*-test (*P < 0.05, ** P < 0.01, *** P < 0.001, **** P < 0.0001, n = 6).

Before initiating *in vivo* verification of the FN1 3'UTR-let-7i-5p-THBS1 axis, we first clarified the direct binding relationship between *THBS1* mRNA and let-7i-5p through a dual-luciferase reporter assay (**Figure S8**). And further experiments were conducted to verify this mechanism *in vivo* (**Figure 7**). The THBS1 expression levels were significantly higher in the metastatic nodules in lungs and tumor nodules in the abdominal cavity of the *FN1* 3′-UTR overexpressed group than that of the negative control group (**Figure 7A** and **7B**). We propose that THBS1 upregulation was caused by competitive enrichment of let-7i-5p by *FN1* 3'-UTR through the ceRNA mechanism. We further applied immunofluorescence assay to detect the co-localizations of *FN1* 3'-UTR and let-7i-5p. A higher proportion of cells co-localized *FN1* 3'-UTR and let-7i-5p in the *FN1* 3′-UTR overexpressed group co-transfected with let-7i-5p than that of the negative control group transfected with let-7i-5p in the metastatic nodules in the lungs (**Figure 7C**-**7D**). The same results were replicated in the sample tissues from the peritoneal implantation assays (**Figure 7E**-**7F**). In order to further illustrate the relationship between let-7i-5p and cell function as well as *FN1* 3'-UTR and *THBS1*, we constructed HGC27 overexpressing let-7i-5p and detected the expression levels of *FN1* 3'UTR and THBS1 in the cells (**Figure S9A-S9C**). Consistent with former results, the expression levels of *FN1* 3'UTR and *THBS1* showed a significant downward trend after let-7i-5p was stably upregulated. Moreover, in the *in vivo* xenograft assays, the speed of tumorigenesis within the lung tissues and the peritoneal cavity of nude mice injected with cells overexpressing let-7i-5p was significantly slower than that in the control group (n = 6 for each group) (**Figure 7G-H and Figure S9D-S9E**).

### *FN1* 3'-UTR signaling exerts oncogenic effects in GC cells

The GSEA results of RNA-seq (**Figure S3E**) were validated to clarify the molecular mechanism by which *FN1* 3′-UTR promotes invasion and metastasis in GC. Mounting evidence suggests that the transformation of GC cells from the epithelial to mesenchymal phenotype results in the loss of polarity and adhesion to homologous cells. This leads to the dissemination of tumor cells and enhances their invasion and migration abilities. TGF-β is a major player in advanced tumor progression that is related to EMT 41, 42. In addition, matrix metalloproteinase 2 (MMP2) and MMP9 are secreted into the ECM and degrade mesenchymal components; their expression levels correlate with aggressive tumor phenotypes in many cancers 43-45. Our immunofluorescence staining assays demonstrated that the EMT-related protein N-cadherin was significantly upregulated, whereas E-cadherin was downregulated in HGC27/AGS-FN1 3′-UTR cells compared with control cells (**Figure 6F**). Epithelial-to-mesenchymal-like phenotypic transformation was confirmed based on the elevated expression of N-cadherin, Slug, and Snail, together with the reduced expression of E-cadherin, ZO1, β-catenin, and CLDN1 (**Figure S10A-S10B**). These results suggest that *FN1* 3′-UTR plays an oncogenic role in GC cells by promoting EMT. Moreover, the upregulated expression of TGFβ1/2 and MMP2/9 in HGC27/AGS-FN1 3′-UTR cells (**Figure 6G-6H**) was accompanied by the induced exocrine secretion of THBS1, TGFβ1/2, and MMP2/9 (**Figure S10C**). This indicates that the oncogenic mechanism of *FN1* 3′-UTR in GC is at least partially attributable to the alterations of EMT-related proteins and the internal and external expression of TGFβ1/2 and MMP2/9. Moreover, EMT induction was significantly higher in cells subjected to *FN1* 3'-UTR overexpression than that in cells overexpressing FN1 protein according to the elevated and decreased expression of N-cadherin and E-cadherin, respectively (**Figure 6F**). Notably, TGFβ1 secretion significantly differed between *FN1* 3'-UTR and FN1 overexpressed cells (**Figure 6G-6H**).

The EMT process was reversed in HGC27/AGS-FN1 3′-UTR cells following THBS1 downregulation (**Figure 6I**). THBS1 is a recognized activator of TGFβ 46-48. Therefore, only the expression relationship of THBS1-MMP2/9 was explored in this study. The downregulation of THBS1 in cells overexpressing* FN1* 3′-UTR inhibited the promotion of MMP2/9 expression and exocrine secretion by* FN1* 3′-UTR (**Figure 6J-6K** and **Figure S11A**). In summary, the *FN1* 3′-UTR in the ceRNA regulatory network might play a role in promoting metastasis mainly by sponging let-7i-5p and upregulating THBS1, as well as by affecting EMT and the expression levels of TGFβ1/2 and MMP2/9 in GC cells to a certain extent. We speculate that THBS1 might be the main factor by which *FN1* 3′-UTR exerts differential oncogenic effects compared with the FN1 protein based on our results and previous studies.

Furthermore, THBS1 and TGFβ1 secretions were significantly higher in cells overexpressing *FN1* 3′-UTR than those in cells overexpressing FN1 protein, while TGFβ2 and MMP2/9 were significantly upregulated in cells overexpressing *FN1* 3′-UTR and FN1 (**Figure S11B**). The difference in TGFβ1 levels was believed to be caused by the variable THBS1 expression between the *FN1* 3′-UTR overexpressing cells and FN1 protein overexpressing cells. We speculated that MMP2/9 upregulation was derived from THBS1 induction in *FN1* 3'-UTR overexpressing cells, whereas it might be associated with the activation of the FAK signaling pathway in FN1 protein overexpressing cells 49. Hence, the mechanism by which the *FN1* 3′-UTR upregulated THBS1 to achieve an oncogenic effect via the ceRNA network was specific to the FN1 protein, and the difference in the expression levels of THBS1 might be the core factor explaining the variations in the clinical significance of FN1 at the RNA and protein levels in GC.

## Discussion

Several molecular mechanisms related to GC initiation and progression are being revealed, owing to advances in specific target identification. For example, ramucirumab (a monoclonal antibody VEGFR2 antagonist) demonstrated significant prognostic improvement in previously treated patients with advanced GC in the RAINBOW trial, while pembrolizumab (which targets immune response-related PD-1) also demonstrated safety and efficacy as a first-line treatment for GC in the KEYNOTE-062 trial 50-53. Several genes and proteins interact with each other and form signaling pathways or regulatory networks that are involved in regulating several physiological and pathological processes, including those in various cancers. We believe that a good target needs to have the following characteristics: outstanding clinical significance, the ability to modulate the molecular mechanism of the tumor, and the ability to play a central role in a cross-linked network. FN1 protein is a glycoprotein mainly expressed in the ECM, and its role in the TME has been thoroughly investigated 23. There is no significant correlation between FN1 protein expression and patient prognosis in ovarian cancer and pancreatic ductal adenocarcinoma 54-56. However, the expression level of FN1 protein is closely related to the degree of tumor infiltration and size in pancreatic ductal adenocarcinoma 55, 56. Our findings revealed that FN1 protein expression did not significantly correlate with OS (*P* = 0.807) in patients with GC. In contrast, Xiao et al. 57 showed that OS (*P* = 0.002) and disease-free survival (DFS) (*P* < 0.001) were significantly lower when FN1 protein expression was higher. The correlation between the FN1 protein expression level and the prognosis or clinicopathology of patients with cancer remains inconclusive, especially in GC. This limits its use as a biomarker or therapeutic target. However, our results revealed the differential clinical significances of FN1 at the mRNA and protein levels. This indicates that FN1 might play a critical oncogenic role at the RNA level in GC. High-throughput sequencing identified the miRNAs competitively sponged by* FN1* 3′-UTR and the targeted genes via the ceRNA mechanism. Finally, a complete ceRNA regulatory network was constructed using *FN1* 3′-UTR as the core. *FN1* 3′-UTR was shown to play a significantly more relevant oncogenic role in GC compared with the FN1 protein.

The selection of therapeutic targets involves considering the clinical significance of the target. Targeted drugs should act on different steps related to these targets and exert anti-cancer effects, such as inhibiting tumor growth, blocking immune escape, and inhibiting tumor invasion and metastasis 51, 53, 58. The 3′-UTR regions of different genes play complex regulatory roles as part of ceRNA networks, and this process affects the phenotypic changes of the tumors. For example, PTEN is a tumor suppressor that harbors several miRNA-binding sites in its 3'-UTR region. The interaction of miRNAs binding to this region leads to the downregulation of *PTEN* expression and activates the PI3K/AKT pathway in various types of cancer, thereby promoting tumor proliferation and inhibiting apoptosis 59. Furthermore, overexpression of *VAPA*, *CNOT6L*, or other genes harboring binding sites for these miRNAs on their 3'-UTR regions competitively sponge miRNAs and block their binding to *PTEN* 3'-UTR via post-transcriptional regulation, resulting in *PTEN* upregulation. Therefore, the tumor suppressive effect of *PTEN* as the core of a ceRNA regulatory network could be strengthened via competitive enrichment of miRNAs and blocking their binding to the 3'-UTR of PTEN. The frequent loss of *PTEN* in melanoma is regulated by the ceRNA mechanism 60. *ZEB2* 3'-UTR acts as a ceRNA to regulate *PTEN* expression via miRNA-based regulation; moreover, *ZEB2* attenuation (which commonly occurs in melanomas) activates the PI3K/AKT pathway by downregulating *PTEN* expression. *CD44* 3'-UTR overexpression inhibits tumor proliferation, angiogenesis, and docetaxel resistance in breast cancer by serving as a competitor to the *CDC42* 3'-UTR for miR-216a, miR-330, and miR-608 binding 61. Therefore, studies elucidating the specific role of 3'-UTR as a link in the ceRNA network are important to further clarify its post-transcriptional regulation. This study revealed the significant effects of *FN1* 3′-UTR on cancer invasion and metastasis and elucidated the complete ceRNA regulatory network related to *FN1* 3′-UTR. Our findings revealed that *FN1* 3′-UTR—as the core of the ceRNA regulatory network and the common target of let-7i-5p, miR-629-5p, miR-423-5p, and miR-296-3p—regulates several oncogenic target genes (such as *THBS1*) to exert oncogenic effects. Notably, many miRNAs in the let-7 miRNA family (including let-7i-5p and let-7a-5p) bound to *FN1* 3'-UTR in the network (**Figure 4B**). The let-7 family was initially identified a few decades ago and acts as a tumor suppressor in lung cancer by reducing the expression of RAS and HMGA2 proteins 62, 63. Furthermore, a significant correlation between the let-7 family and HMGA2 was noted in GC 64. Several studies show that the overexpression of LIN28 (a let-7 family negative regulator that blocks the conversion of let-7 precursors to mature miRNAs) results in malignant phenotypic changes in various tumors 65-67. Furthermore, HMGA2, LIN28, and IGF2BP1 (which have a binding relationship with the let-7 family) form an oncogenic triangle and exert synergistic effects on carcinogenesis. For example, LIN28 inhibits let-7 expression and blocks the targeting effect of let-7 and HMGA2 or IGF2BP1 to exert an oncogenic effect 68. This study showed that FN1 3'-UTR is targeted by the let-7 family and promotes the invasion and metastasis of GC cells. This supports the tumor suppressor role of the let-7 family. In addition, THBS1 is a key factor playing a pivotal role in mediating the oncogenic effects of *FN1* 3′-UTR via the ceRNA regulatory network and is a target of let-7i-5p. Therefore, it has garnered significant interest for its role in tumors. THBS1 may inhibit cancer development by suppressing angiogenesis 69-71. However, THBS1 also acts as a transcriptional activator of TGF-β to activate the TGF-β signaling pathway, thus promoting cancer development 72. The TGF-β signaling pathway-promoting effect of THBS1 might also be the reason for the stronger oncogenic effect of *FN1* 3'-UTR compared with the FN1 protein based on differences in the secretion levels of TGFβ1/2 in cells overexpressing *FN1* 3′-UTR and FN1 protein in this study. THBS1 can bind to CD47 in hematological tumors, thereby inhibiting the immune response to cancer cells 73. Moreover, it regulates the fibrosis of peritoneal mesothelial cells via activating the TGFβ1/Smad3 signaling pathway and inducing the transformation of mesothelial cells to a mesenchymal cell phenotype 74. Collectively, these results support our conclusion that GC cells that highly express THBS1 are more likely to undergo peritoneal metastasis via interaction with mesothelial cells.

**Figure 8:**
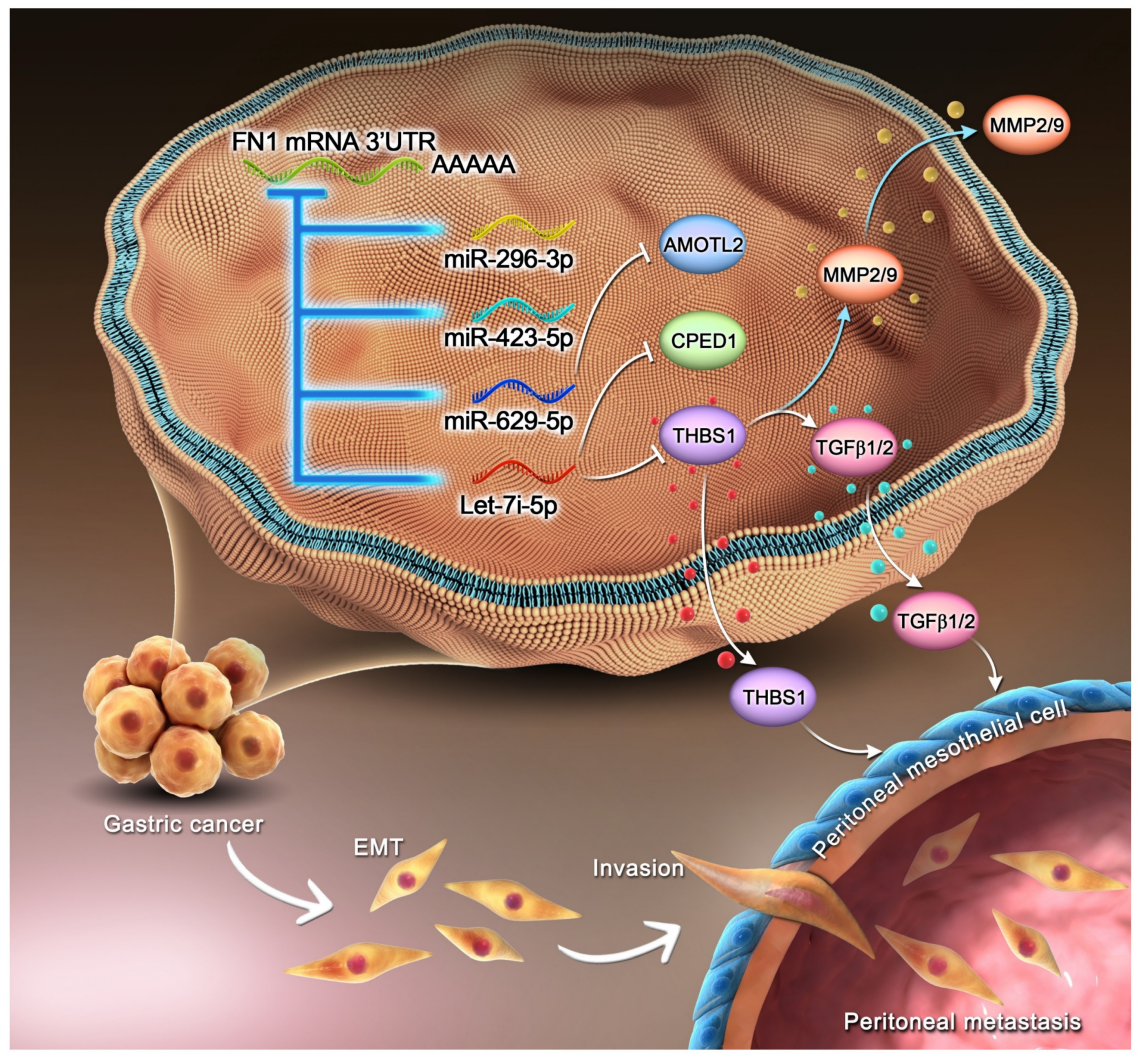
Mechanism of the regulatory network and function of the *FN1* 3′-UTR region.

Our findings highlight the existence of a regulatory network wherein* FN1* 3′-UTR acts as the core element, affects the invasion and metastasis of GC cells, and modulates the TME (**Figure 8**). Furthermore, correlation of *FN1* 3′-UTR with metastasis-related molecular mechanisms and the more aggressive nature of *FN1* 3′-UTR compared with the FN1 protein suggest that *FN1* 3′-UTR might be a better target for inhibiting cancer development than the FN1 protein. Our future studies will explore intracellular transcriptomic changes induced by overexpressing the *FN1* CDS region using RNA-seq. Combining with the data obtained in this study, further analysis of the differences of molecular mechanisms between FN1 3'-UTR and CDS regions will provide further evidence to screen potential therapeutic targets.

In summary, a ceRNA regulatory network was constructed and validated in GC using *FN1* 3′-UTR as its core via high-throughput sequencing. The upregulation of *FN1* 3′-UTR expression resulted in more aggressive GC cells. Thus, *FN1* 3′-UTR might exert a stronger oncogenic effect than FN1 protein by sponging let-7i-5p to regulate *THBS1* expression. These findings imply that* FN1* 3′-UTR is a potential therapeutic target for the clinical management of GC.

## Materials and methods

### Gastric cancer patient specimens and bioinformatic datasets

Two independent cohorts containing 222 and 102 GC patients that underwent radical surgery at the First Hospital of China Medical University (CMU) were included in this study. CMU cohort-1 included 222 patients diagnosed with GC in 2008 that were followed up until 2015. CMU cohort-2 included the clinical information of 102 patients retrieved from our previously published study 15. The previously determined protein expression levels of FN1 in the GC tissues of these patients were further analyzed in this study. The study involving human participants was reviewed and approved by the Ethics and Indications Committee of CMU for clinical investigation (approval number: [2017]2017-59-2).

The RNA expression profiles and clinical data of TCGA-STAD and ACRG cohorts were downloaded from TCGA database and GSE62254 dataset in the Gene Expression Omnibus (GEO) database, respectively, for bioinformatic analyses.

### Tissue microarray slides

The tissues were fixed in 10% formalin overnight and embedded in paraffin. Then, hematoxylin and eosin staining was performed to locate the representative region of solid tumor tissue and normal tissue (at least 5 cm away from the cancer lesions). Tissue microarray (TMA) slides were made by a manual tissue arraying tool and a donor block was prepared by punching out a 1.5-mm diameter tissue core and transferring it to the recipient block. The 4-µm-thick sections were cut from the TMA block strictly and continuously.

### ISH

An ISH assay for* FN1* mRNA was performed on TMAs according to the manufacturer's protocol (Boster Biological Technology, Wuhan, China). Then, 4-mm sections were hybridized with mixed Digoxin (DIG)-modified DNA probes targeting *FN1* 3'-UTR: 1 5′-GCATGATCTTGTTACTGTGATATTTTAAATATCCACAGTA-3′; 2 5′-ATTTACATTCCACAACTTGAAGTTCATCTATTTGATATAA-3′; 3 5′-CAATATTTATTAAAATTGCTAGTTTACCGTTCAGAAGTAT-3′. The following DNA probes targeted the *FN1* CDS region: 1 5′-GCAGACCACATCGAGCGGATCTGGCCCCTTCACCGATGTT-3′; 2 5′-CTGGGCAACGGAGTCAGCTGCCAAGAGACAGCTGTAACCC-3′; 3 5′-ATTAATTACCGAACAGAAATTGACAAACCATCCCAGATGCA-3′. Two experienced pathologists independently scored the results based on the intensity and proportion of positive cells. Specific evaluation methods were elaborated in the following content.

### Immunohistochemistry

Immunohistochemistry (IHC) was mainly applied to verify the expression of THBS1 in tumor nodules from *in vivo* xenograft assays. Paraffin sections were deparaffinized in xylene and hydrated in citrate buffer (pH 6.0); antigen retrieval was mediated by heating the samples in a water bath for 15 min. These TMAs were treated with 3% hydrogen peroxide for 15 min at room temperature and incubated with 10% goat serum. The sections were incubated overnight at 4°C with anti-THBS1 antibody (1:200, 18304-1-AP, Proteintech Group, IL, USA), followed by incubation with biotin-labeled secondary antibody at room temperature for 30 min, and stained with 3,3′-diaminobenzidine (DAB) for 1-3 min under microscopic control. The TMAs were counterstained with hematoxylin, dehydrated, rinsed, and mounted. The negative controls were stained without primary antibody.

### Evaluation of ISH and IHC staining

The immunoreactivities of *FN1* 3'-UTR, *FN1* CDS in ISH, and THBS1 in IHC were evaluated in an unbiased manner without patients' information, based on the staining intensity and the proportion of positive cells. The staining intensity of FN1 3'-UTR, for example, was scored as 0 (-) negative, 1 (+) weak, 2 (++) moderate, and 3 (+++) strong. Then, the proportion of positive cells was considered as follows: 0, negative; 1, positive cells ≤10%; 2, positive cells >10% and ≤50%; 3, positive cells >50% and ≤75%; and 4, positive cells >75%. The two scores were multiplied, and the expression was graded as follows: negative, score = 0 (-); weak expression, score = 1-4 (+); moderate expression, score = 5-8 (++); and strong expression, score = 9-12 (+++). Each specimen score of the staining was analyzed by the Youden index to evaluate the prognosis and determine the optimum cutoff of the ISH positivity threshold. Finally, these specimens were divided into two groups according to their scores: 0-4 represents the low expression group, and 5-12 represents the high expression group for both *FN1* 3'-UTR and *FN1* CDS. The IHC scores of THBS1 were in tumor nodules from *in vivo* xenograft assays were used to compare the difference between *FN1* 3′-UTR overexpressed and negative control groups by Student's *t*-test.

### Cell lines and cell culture

Human GC cell lines SGC7901, MGC803, HGC27, AGS, and MKN45 were purchased from the Cell Bank of the Type Culture Collection of the Chinese Academy of Sciences (Shanghai, China). Human GC cells were cultured in DMEM, RPMI 1640, or F12/DMEM supplemented with 10% fetal bovine serum (FBS). A human peritoneal mesothelial cell line (HMrSV5) established by Prof. Ronco 75 was kindly provided by Prof. You‐Ming Peng (Second Hospital of Zhongnan University, Changsha, China). HMrSV5 cells were cultured in RPMI 1640 medium supplemented with 10% FBS. All cell cultures were incubated in a 5% CO_2_ atmosphere at 37°C. The indirect coculture between GC cell and HMrSV5 cells was performed using Transwell chambers (0.4-μm pore size, Corning, NY, USA) separated by a polycarbonate membrane 76, 77. GC cells (5.0 × 10^5^) and HMrSV5 cells (1.0 × 10^5^) were seeded into the upper and bottom chambers, respectively. The GC cells had no direct contact with HMrSV5 cells; however, soluble factors derived from the GC cell lines could reach the HMrSV5 cells.

### Transfection of plasmids, miRNAs, and siRNAs

Transfections were performed using Lipofectamine 3000 reagent (Thermo Fisher Scientific) according to the manufacturer's protocol. The pcDNA3.1 vector for overexpressing *FN1* 3′-UTR or CDS (NM_002026.4) was obtained from GeneChem (Shanghai, China). The siRNAs were obtained from Santa Cruz Biotechnology (CA, USA). The miRNA mimics and negative controls were obtained from GenePharma (Suzhou, China). The miRNAs were transfected at a final concentration of 50 nM. Cells were collected 48 h after transfection for further analyses. The sequences of the siRNAs and miRNA mimics are listed in **Table S24**.

### Construction of stable overexpression cell lines

HGC27 and AGS cells were infected with lentiviral particles and screened with puromycin at a final concentration of 10 μg/mL to construct stable *FN1* 3′-UTR overexpressed cells. Puromycin-resistant cells were collected after six days and verified by quantitative real-time polymerase chain reaction (qRT-PCR). Stable FN1 protein-overexpressing cells for *in vivo* xenograft assays were prepared by transfecting HGC27 cells with pcDNA-FN1 and screened using puromycin at a final concentration of 5 μg/mL. *In vivo* imaging involved infecting HGC27-FN1 3′-UTR, HGC27-FN1, and HGC27-control cells with luciferase-lentiviral vectors obtained from GeneChem (Shanghai, China). G418 was used to screen the infected cells at a final concentration of 300 μg/mL, hereafter referred to as HGC27-FN1 3′-UTR-Luc, HGC27-FN1-Luc, and HGC27-Control-Luc cells, respectively. Following the same method, we also constructed HGC27 cell lines that stably overexpressing let-7i-5p and contain a luciferase tag, along with a negative control cell line, hereafter referred to as HGC27-let-7i-5p and HGC27-Control cells, respectively.

### Western blotting analysis

Total protein was isolated from cells using RIPA lysis buffer (Beyotime). Western blot analysis was performed as previously described 15. The intensity of the protein bands was quantified using ImageJ software (NIH). Primary antibody against FN1 (1:1000, ab32419) was purchased from Abcam (Shanghai, China). Antibodies against THBS1 (1:1000, 18304-1-AP), CPED1 (1:1000, 20924-1-AP), AMOTL2 (1:1000, 23351-1-AP), TGFβ1 (1:1000, 21898-1-AP), TGFβ2 (1:1000, 19999-1-AP), and GAPDH (1:10000, 60004-1-Ig) were purchased from Proteintech Group (IL, USA). Antibodies against E-cadherin (1:500, #3195), N-cadherin (1:500, #13116), Slug (1:1000, #9585), Snail (1:1000, #3879), ZO1 (1:1000, #8193), β-catenin (1:1000, #8480), CLDN1 (1:1000, #13255), MMP2 (1:1000, #40994), and MMP9 (1:1000, #13667) were purchased from Cell Signaling Technology (MA, USA).

### RNA isolation and qRT-PCR

Total RNA was isolated using TRIzol reagent (Invitrogen, CA, USA). Total RNA (2 μg) was used to synthesize first-strand cDNA using a PrimeScript^TM^ Reverse Transcription Reagent Kit (Takara Biomedical Technology Co., Ltd., China). Next, qRT-PCR was performed using SYBR Green mix (Applied Biosystems) with a 7500 Fast Real-Time PCR system (Applied Biosystems) according to the manufacturer's instructions. The primer sequences are listed in **Table S24**.

### Cell migration and invasion assays

Cell migration assays were performed in a Transwell chamber (8-μm pore size, Corning, NY, USA), and the invasion assay was performed using a Transwell chamber supplemented with Matrigel (BD Biosciences, NJ, USA). The cells were resuspended in serum-free medium (5 × 10^4^ cells/mL) and placed in the upper chamber. The bottom chamber was filled with 600 μL culture medium containing 10% FBS, and the plate was cultured in a 5% CO_2_ supplemented incubator set at 37°C. Cells that moved to the bottom chamber were removed, fixed with 75% ethanol, and stained with 4% trypan blue (Solarbio, Beijing, China). Cells that migrated or invaded were counted under a microscope and reflected the migration or invasion ability.

### Wound-healing assay

Cells (5 × 10^4^) were seeded in six-well plates and allowed to grow to 90-100% confluence. A wound was created in the cells using a 20-200 μL sterile pipette tip, the wounded monolayer was washed with PBS to remove cell debris, and serum-free medium was used to maintain the cells. Wound images were taken at 0, 12, and 24 h and the rate of wound closure within 24 h was considered representative of the surface mobility.

### Cell adhesion assay

The cells (1×10^5^ cells/well) were seeded in 96-well plates. The medium was aspirated, the cells were washed with PBS, and stained with 0.1% crystal violet (Solarbio, Beijing, China) after 30 min and 60 min. The adhered cells reflecting cellular adhesion ability were counted under a microscope 78.

### RNA pull-down assays

RNA pull-down assays were performed using HGC27-FN1 3′-UTR and AGS-FN1 3′-UTR cells and the Pierce Magnetic RNA-Protein Pull-Down kit (Thermo Fisher Scientific, MA, USA) according to the manufacturer's protocol before performing miRNA-seq. *In vitro* transcribed (IVT) RNA positive and negative probes to the *FN1* 3′-UTR were used in the pull-down assays and prepared with the AmpliScribe™ T7 High Yield Transcription kit (Epicenter, WISC, USA). Briefly, 1.0 × 10^7^ cells were collected and washed with PBS. The cells were lysed using 1 mL RIPA lysis buffer and centrifuged at 12000 x *g* for 15 min at 4°C to collect the supernatant. Next, 50 μL washed streptavidin magnetic beads were incubated with 5 μg biotinylated IVT antisense RNA for 30 min at room temperature with agitation. The probe-coated beads were then incubated with 500 μL cell lysis supernatant for 1 h. The beads were washed five times with wash buffer and then eluted.

### High-throughput sequencing and data analysis

HGC27-FN1 3′-UTR and HGC27-control cells were prepared for transcriptome sequencing. Total RNA was isolated using TRIzol reagent (Invitrogen, CA, USA). Gene-specific or random primers were used to generate cDNA. A strand-specific RNA sequencing library was prepared using the KC-Digital^TM^ Stranded mRNA Library Prep kit for Illumina^®^ after quantitative analysis and quality inspection. RNA-seq was performed using the Illumina Nova 6000 sequencing system (Wuhan SeqHealth Co., Ltd., Wuhan, China). Reads per kilobase per million reads (RPKM) were calculated as gene expression levels after data preprocessing 79.

RNA pull-down assays were performed using HGC27-FN1 3′-UTR and AGS-FN1 3′-UTR cells, and the bound RNA in the pull-down materials was purified for miRNA sequencing. The library was denatured to generate single-stranded DNA molecules, captured on Illumina flow cells, amplified *in situ* as clusters, and sequenced on an Illumina Novaseq 6000 sequencing system (Cloud-Seq Biotech Ltd. Co., Shanghai, China) after quantitative analysis and quality inspection. The number of mature miRNA-mapped tags was defined as the raw miRNA expression level. The read counts were normalized using a tag counts per million-aligned (TPA) miRNAs approach 80. The sequencing data were deposited in the NCBI GEO datasets under accession number GSE197424.

### Coculture, invasion, and adhesion assays

The adhesion and invasion abilities of GC cells were assessed in the presence of HMrSV5 cells as previously described 77. Invasion assays involved seeding HMrSV5 cells into the upper chamber of Transwell inserts (8-μm-pore size, Corning, NY, USA) coated with 100 μL Matrigel. Once the HMrSV5 cells reached 90% confluence, 5 × 10^4^ GC cells were resuspended in 100 μL serum-free medium and added to the top chamber. The bottom chamber was filled with 600 μL medium containing 10% FBS, and the plate was incubated at 37°C supplemented with 5% CO_2_. The chamber was removed after cells migrated to the bottom chamber. These cells were fixed with 75% ethanol and stained with 4% trypan blue (Solarbio, Beijing, China). The invading cells were counted under a microscope and reflected the invasion-resistance ability of HMrSV5 cells. Adhesion assays involved coculturing HMrSV5 cells with GC cells in a 96‐well plate. HMrSV5 cells were cultured in a 96-well plate until 90 % confluence. The GC cells were incubated with 5 μmol/L Calcein-AM (Sigma-Aldrich, MO, USA), added to the 96‐well plate, and incubated for 1 h. Then, the plates were washed with PBS to remove the non-adherent GC cells. The adherent cells were counted under a fluorescence microscope and reflected the adhesion ability of HMrSV5 cells.

### *In vivo* xenograft assays

Metastasis assays involved injecting HGC27-FN1 3′-UTR-Luc, HGC27-FN1-Luc, and HGC27-Control-Luc cells (2 × 10^6^ cells) as well as HGC27-let-7i-5p and HGC27-Control cells (2 × 10^6^ cells) into the tail vein of 4-week-old female BALB/c nude mice randomly categorized into three groups (n = 6 for each group). All animals were subjected to *in vivo* optical imaging and euthanized via cervical dislocation after nine weeks. Next, lung tissues were removed and embedded in paraffin for hematoxylin and eosin staining. Peritoneal implantation assays involved injecting HGC27-FN1 3′-UTR-Luc, HGC27-FN1-Luc, and HGC27-Control-Luc cells (2 × 10^6^ cells) as well as HGC27-let-7i-5p-Luc and HGC27-Control-Luc cells (2 × 10^6^ cells) into the abdomen of 4-week-old female BALB/c nude mice randomly categorized into three groups (n = 6 for each group). All animals were subjected to *in vivo* optical imaging and euthanized via cervical dislocation after 5 weeks. The abdominal cavity of nude mice was opened to observe tumor formation, and the tumor nodules were removed and weighed. The Animal Ethics Committee of the CMU approved all animal experiments [IACUC Issue No. CMU2021628].

### Luciferase reporter assay

Luciferase reporters were generated using the pMIR-REPORTER vector (GENEWIZ, NJ, USA). The complete 3′-UTRs of human *FN1* mRNA (NM_002026.4) and *THBS1* mRNA (NM_003246.4) were amplified and cloned into the pMIR-REPORTER vector to construct pMIR-REPORTER-*FN1* 3′-UTR and pMIR-REPORTER-*THBS1* 3′-UTR. The luciferase reporter was co-transfected with let-7i-5p, miR-629-5p, miR-423-5p, miR-296-3p, let-7i-5p-mut, let-7i-5p-mut-2, miR-629-5p-mut, miR-423-5p-mut, miR-296-3p-mut, or miR-NC into cells using Lipofectamine 3000 reagent according to the manufacturer's protocol. The relative luciferase activity was measured using the Dual-Luciferase Reporter Assay system (Promega, Madison, WISC, USA) on an Infinite M200 PRO microplate reader (Tecan, Shanghai, China).

### RNA-binding protein immunoprecipitation

HGC27-FN1 3′-UTR, HGC27-control, AGS-FN1 3′-UTR, AGS-control cells, anti-AGO2 antibody (Millipore, MA, USA), and the Magna RIP^TM^ RNA-Binding Protein Immunoprecipitation kit (Millipore, MA, USA) were used for RIP experiments according to the manufacturer's protocol. RNA was isolated from the immunoprecipitates and quantified using a NanoPhotometer UV spectrophotometer (Thermo Fisher Scientific, MA, USA). Finally, qRT-PCR was performed to examine the expression levels of *FN1* 3′-UTR, *THBS1*, *CPED1*, and *AMOTL2*.

### Immunofluorescence assay

The cells (5 × 10^4^) were seeded onto glass coverslips in six-well plates and incubated for 24 h. Cells were fixed in 4% paraformaldehyde and blocked with bovine serum albumin. The cells were incubated overnight at 4°C with primary antibodies against E-cadherin (1:200) and N-cadherin (1:100), washed with PBS, and incubated with secondary antibodies (Cy3 conjugated donkey anti-mouse IgG, 1:200, Servicebio, Wuhan, China; FITC-conjugated goat anti-mouse IgG, 1:100, Servicebio, Wuhan, China). DNA was stained with 4′,6-diamidino-2-phenylindole (DAPI) (Sigma-Aldrich, St. Louis, MO, USA). Confocal scanning was performed using an Ultraview Vox Spinning Disc confocal microscope (PerkinElmer, MA, USA). The fluorescence intensity of each primary color channel was measured using ImageJ software and the data was illustrated as the mean fluorescence intensity (MFI). A Student's *t*-test was used to compare the MFIs of different indexes.

### Fluorescence *in situ* hybridization (FISH)

FISH was performed in the paraffin sections of tumor nodules from *in vivo* xenograft assays using a commercial kit from Servicebio (Wuhan, China) according to the manufacturer's protocol. Sections were blocked by goat serum at 37°C for 1 h, and incubated at 4°C overnight with the primary cDNA oligonucleotide probes targeting either *FN1* 3'-UTR or let-7i-5p. Then, the sections were incubated with secondary antibody conjugated with green or red dye for 1 h. The nucleus was stained using DAPI (Sigma-Aldrich, St. Louis, MO, USA) for 15 min. Samples were observed using an Ultraview Vox Spinning Disc confocal microscope (PerkinElmer, MA, USA).

The sequences of the probe targeting *FN1* 3'-UTR were as follows: 1 5′-AGACATGCTTGTTCCTCTGGATTGG-3′; 2 5′-AAGCTGGGTCTGCTAACATCACTCC-3′; 3 5′-GAGCAAAGGGCTTAAGAAAGAAAGAAG-3′; 4 5′-GTTGAGCTGAAGCTGGAGAACTTCC-3′; 5 5′-CAGCCCTCATTTATGAGAAAACCCTC-3′. The sequence of the probe targeting let-7i-5p was 5′-AACAGCACAAACTACTACCTCA-3′.

### Statistical analyses

Categorical data of clinicopathological characteristics were compared using Pearson's chi-square test or Fisher's exact test. Student's *t*-test and Wilcoxon signed-rank test were performed to compare differences in continuous data distribution between the two groups. The data were analyzed using the Kaplan-Meier method with log-rank test, Spearman's correlation analysis, and Cox multivariate analysis. All statistical analyses were performed using R (version 3.6.0; R Foundation for Statistical Computing, Vienna, Austria) and SPSS software (version 23.0; IBM Corp, Armonk, NY, USA). A two-tailed *P* < 0.05 indicated statistical significance. *P < 0.05 was considered statistically significant, and ** P < 0.01, *** P < 0.001, and **** P < 0.0001 were considered highly significant.

## Supplementary Material

Supplementary figures.

Supplementary tables.

## Abbreviations

GC: gastric cancer
3'-UTR: 3′-untranslated region
OS: overall survival
TKI: tyrosine kinase inhibitor
miRNA: microRNA
FN: fibronectin
ECM: extracellular matrix
CAFs: cancer-associated fibroblasts
TME: tumor microenvironment
GEPIA: Gene Expression Profile Interactive Analysis
ACRG: Asian Cancer Research Group
CDS: coding sequence
ISH: *in situ* hybridization
CMU: Chinese Medical University
HR: hazard ratio
CI: confidence interval
DEG: differentially expressed gene
GO: Gene Ontology
KEGG: Kyoto Encyclopedia of Genes and Genomes
TGFβ: transforming growth factor β
GSEA: Gene Set Enrichment Analysis
EMT: epithelial-mesenchymal transition
ceRNA: competing endogenous RNA
lncRNA: long noncoding RNA
circRNA: circular RNA
miR-seq: miRNA-sequencing
RIP: RNA immunoprecipitation chip
siRNA: small interfering RNA
MMP: matrix metalloproteinase
DFS: disease-free survival
GEO: Gene Expression Omnibus
TMA: tissue microarray
IHC: immunohistochemistry
DAB: 3,3′-diaminobenzidine
FBS: fetal bovine serum
qRT-PCR: quantitative real-time polymerase chain reaction
IVT: *in vitro* transcribed
RPKM: reads per kilobase per million reads
TPA: tag counts per million-aligned
MFI: mean fluorescence intensity
FISH: fluorescence in situ hybridization
