# Supplementary material for: FN1 mRNA 3'-UTR supersedes traditional fibronectin 1 in facilitating the invasion and metastasis of gastric cancer through the FN1 3'-UTR-let-7i-5p-THBS1 axis
Source: Theranostics. 2023 Sep 25;13(14):5130–50. doi: 10.7150/thno.82492 (PMC10526670; doi:10.7150/thno.82492)
Supplement: Supplementary file 1 — Supplementary figures. [file thnov13p5130s1.pdf]

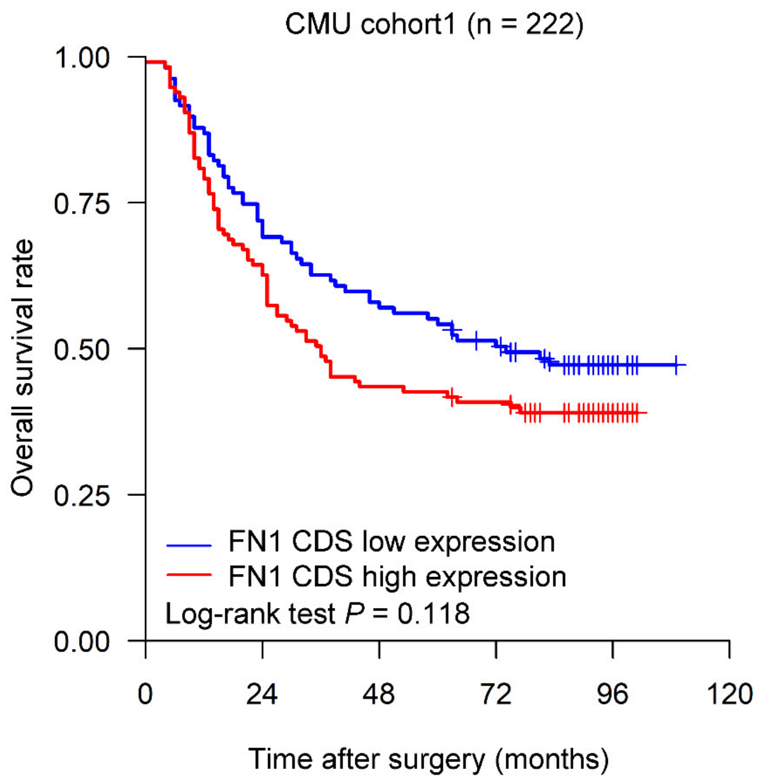

Figure S1. Kaplan-Meier curve of low and high expression of *FN1* CDS in CMU cohort 1.

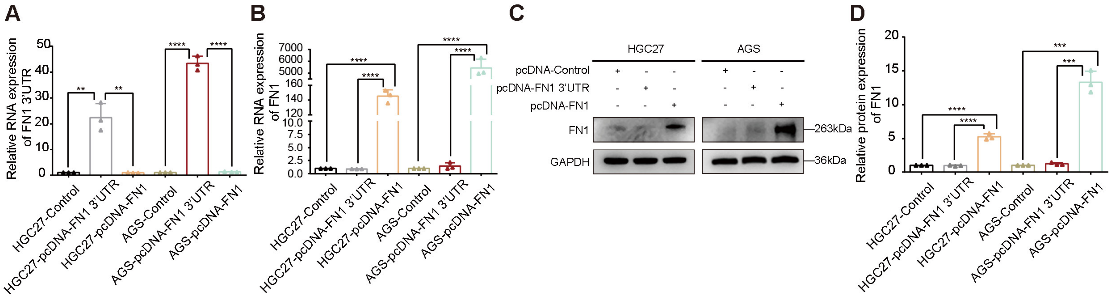

Figure S2. Real-time PCR shows the relative expression levels of *FN1* 3′- UTR and *FN1* mRNA in HGC27 and AGS cells overexpressing indicated genes (A, B). The relative protein levels of FN1 and corresponding analysis were determined by western blot and ImageJ software using HGC27 and AGS cells (C, D). The data are presented as a histogram of the mean ± SEM of three independent experiments in A, B and D and compared by Student's *t*-test (**P* < 0.05, ** *P* < 0.01, *** *P* < 0.001, **** *P* < 0.0001, n = 3).

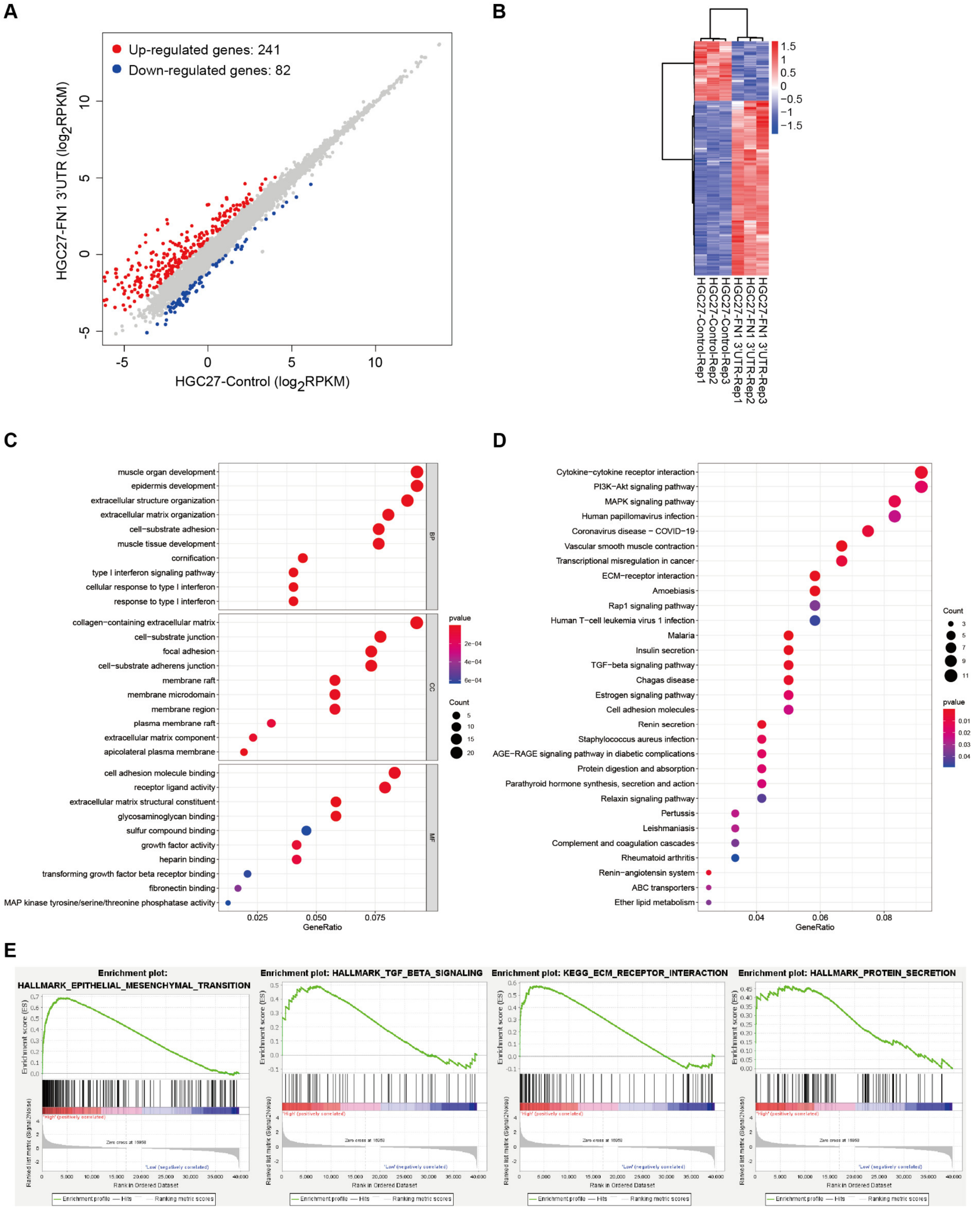

Figure S3. Scatter plot (A) and heatmap (B) of differentially expressed genes (DEGs) in *FN1* 3′-untranslated region (UTR)-stable overexpressed HGC27 cells compared with the negative control cells. Gene Ontology enrichment (C) and Kyoto Encyclopedia of Genes and Genomes pathway (D) analyses of the 323 DEGs were performed. Gene Set Enrichment Analysis of RNA-seq data revealed the *FN1* 3′-UTR affected pathways.

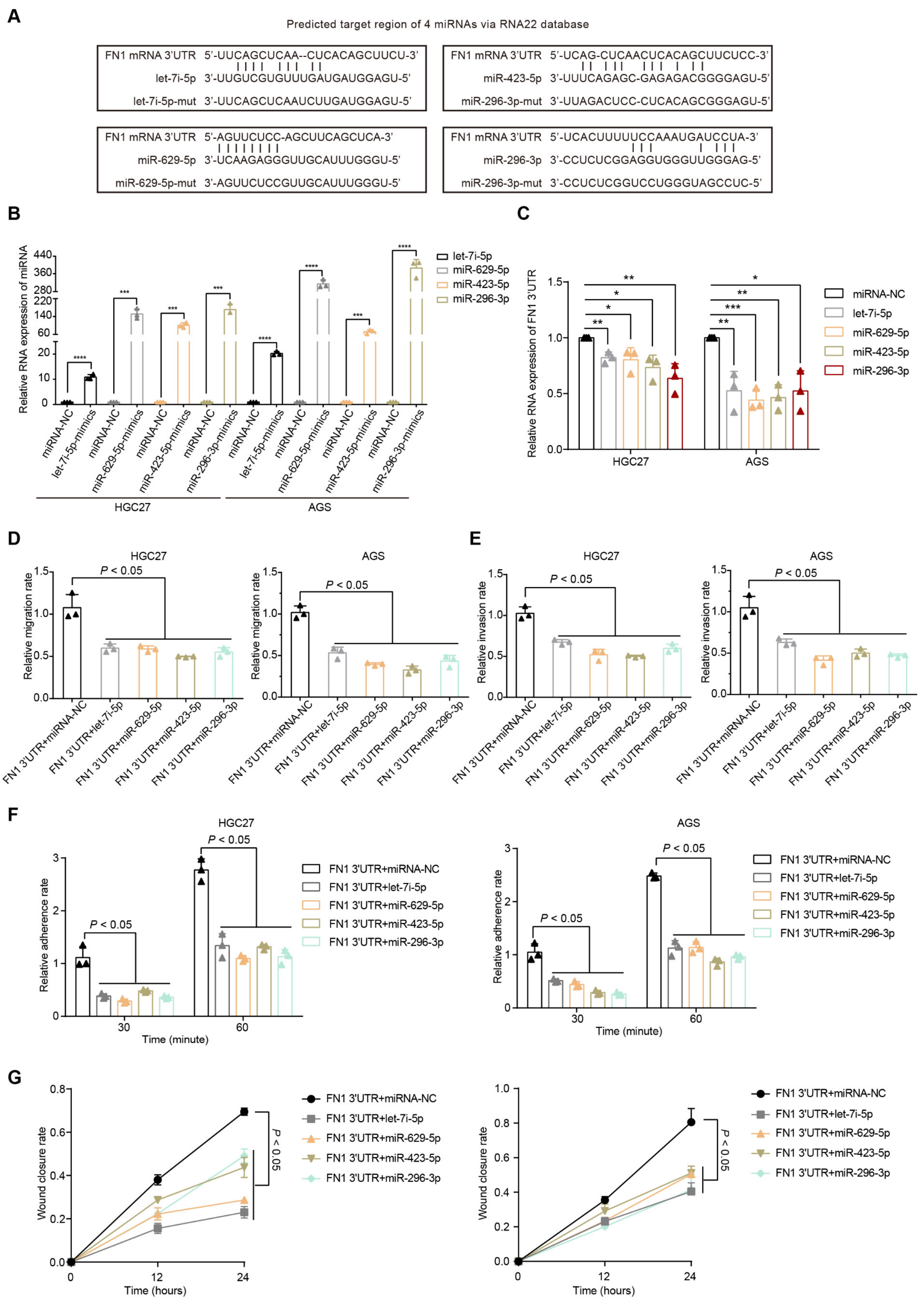

Figure S4. RNA22 database predicted binding sites for let-7i-5p, miR-423-5p, miR-629-5p and miR-296-3p on the *FN1* 3′-untranslated region (UTR) (A). The transfection efficiencies of indicated miRNA mimics in HGC27 and AGS cells were validated by real-time PCR (B). The relative expressions of *FN1* 3′-UTR were determined

by real-time PCR after transfection with indicated miRNA mimics in HGC27 and AGS cells (C). The relative migration (D) and invasion (E) rate, determined by ImageJ software, of *FN1* 3′-UTR affected by indicated miRNAs in HGC27 and AGS cells. The relative adherence rate, determined by ImageJ software, of *FN1* 3′-UTR regulated by indicated miRNAs within 30min or 60min in HGC27 and AGS cells (F). The wound closure rate, determined by ImageJ software, of *FN1* 3′-UTR altered by indicated miRNAs in HGC27 and AGS cells (G). The data are presented as a histogram of the mean ± SEM of three independent experiments in B-G and compared by Student's *t*-test (*$P < 0.05$, ** $P < 0.01$, *** $P < 0.001$, **** $P < 0.0001$, n = 3).

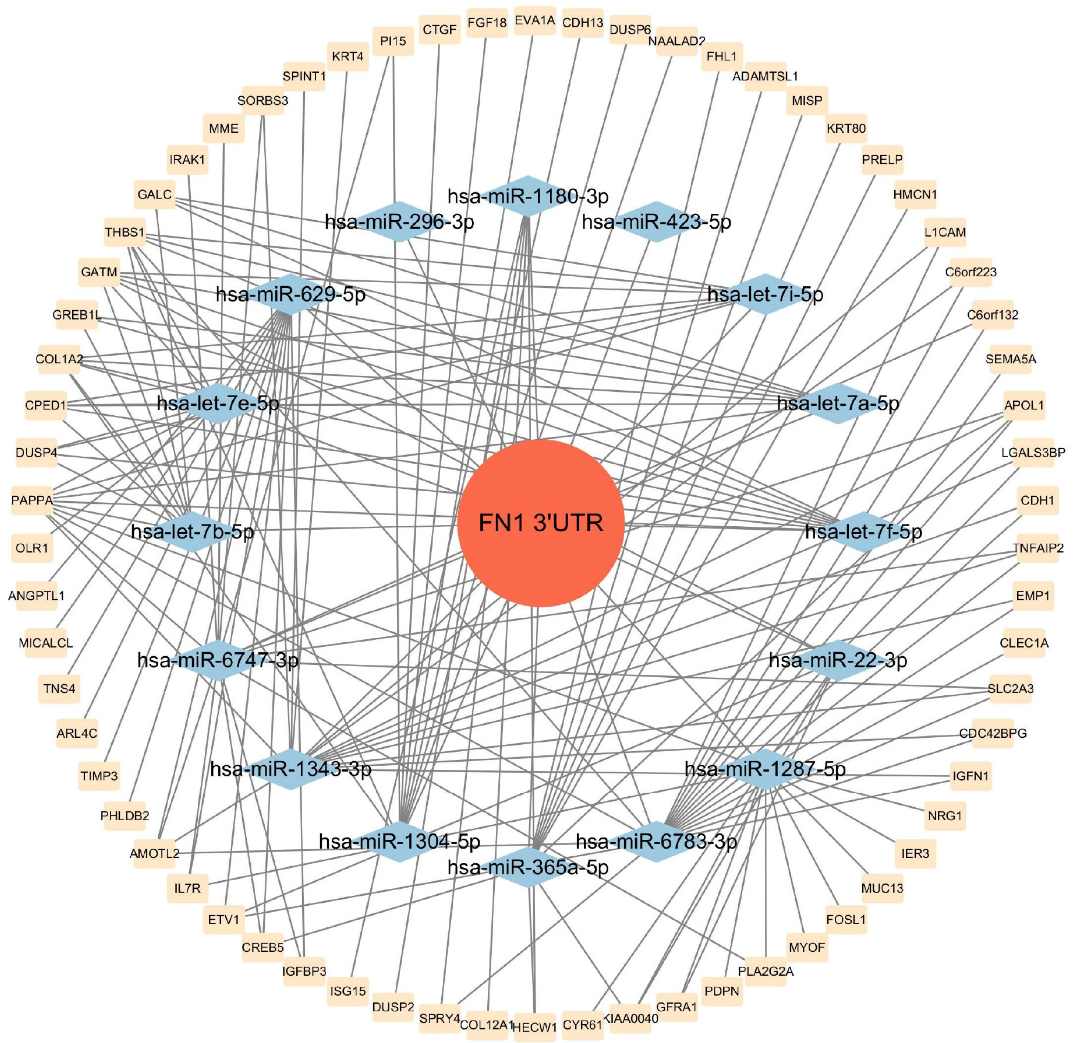

Figure S5. The whole ceRNA regulatory network using *FN1* 3′-UTR as the core.

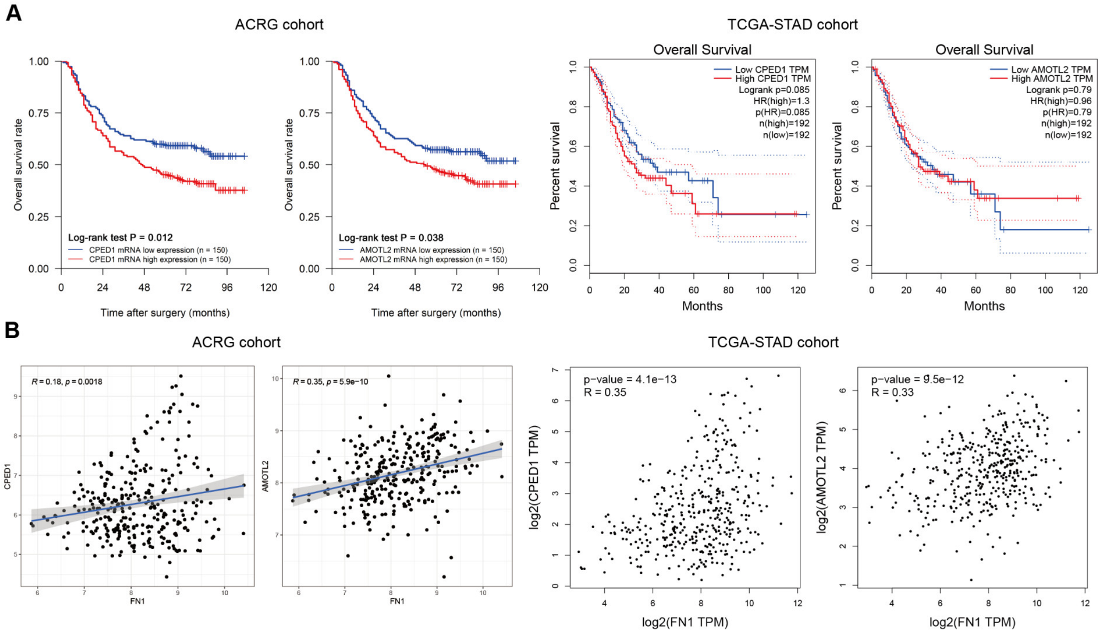

Figure S6. Kaplan-Meier curves of low and high expression of *CPED1* and *AMOTL2* in the ACRG and TCGA-STAD cohorts (A). The correlations between mRNA expressions of *CPED1*, *AMOTL2*, and *FN1* in the ACRG and TCGA-STAD cohorts (B).

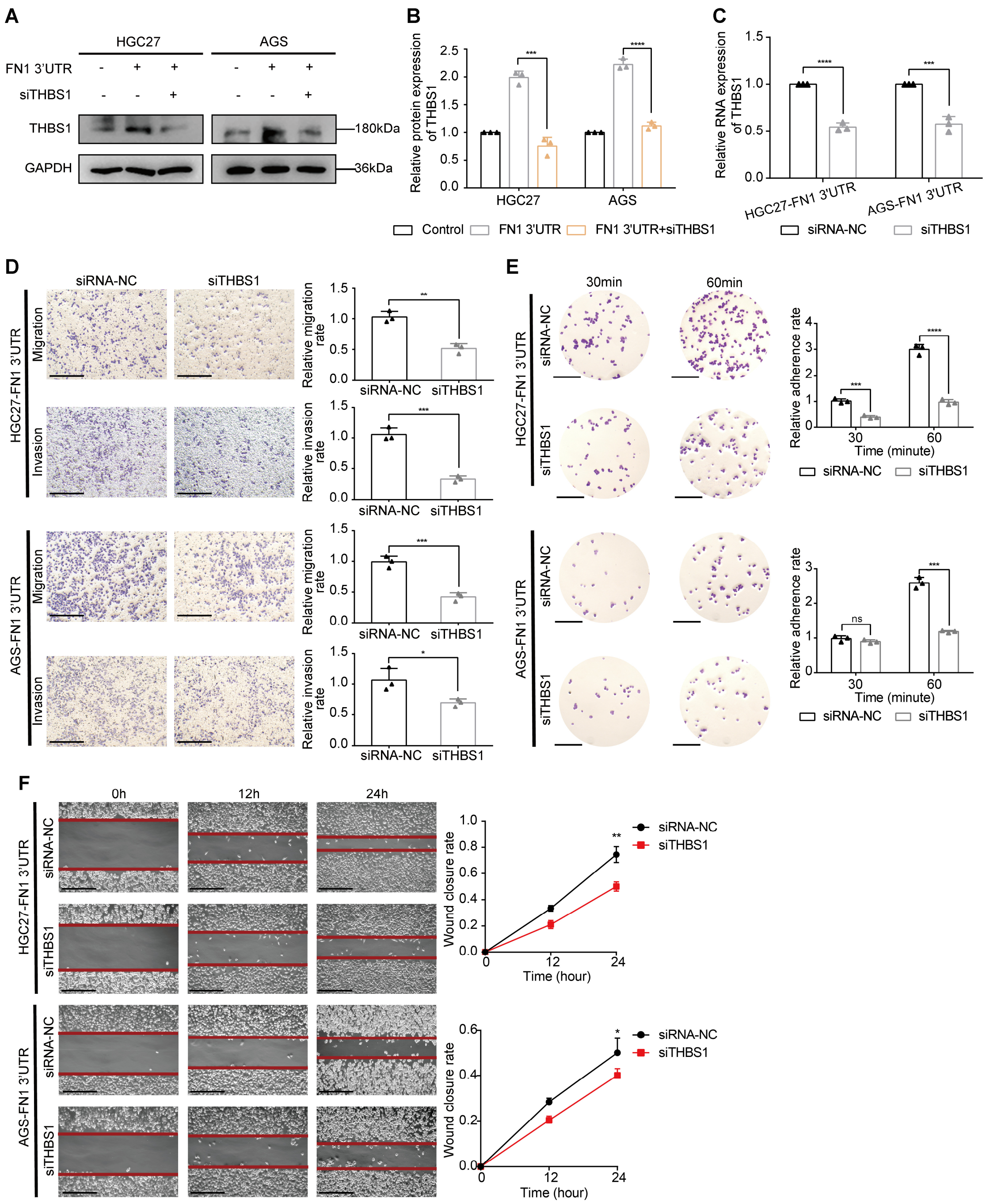

Figure S7. The transfection efficiency of siTHBS1 in *FN1* 3′-untranslated region (UTR) overexpressed HGC27 and AGS cells were validated by western blot and real-time PCR analyses (A, B, C). The migration and invasion abilities of *FN1* 3′-UTR overexpressed cells after silencing THBS1. Scale bars = 500μm (D). The adhesion ability within 30min or 60min (scale bars = 1000μm) (E) and surface mobilities (scale bars = 500μm) (F) of the *FN1* 3′-UTR overexpressed group after silencing THBS1. The data are presented as a histogram of the mean ± SEM of three independent experiments in B-F and compared by Student's *t*-test (**P* < 0.05, ** *P* < 0.01, *** *P* < 0.001, **** *P* < 0.0001, n = 3).

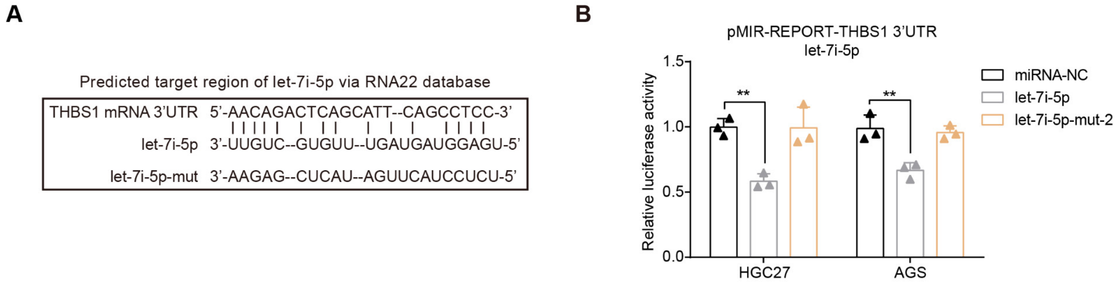

Figure S8. RNA22 database predicted binding sites for let-7i-5p on the *THBS1* 3′-untranslated region (UTR) (A). Luciferase activity of the *THBS1* 3′-UTR in GC cells co-transfected with the mimics of let-7i-5p or let-7i-5p mut-2. Data are presented as the relative ratio of Renilla luciferase activity (B). The data are presented as a histogram of the mean ± SEM of three independent experiments and compared by Student's *t*-test (*P < 0.05, ** P < 0.01, *** P < 0.001, **** P < 0.0001, n = 3).

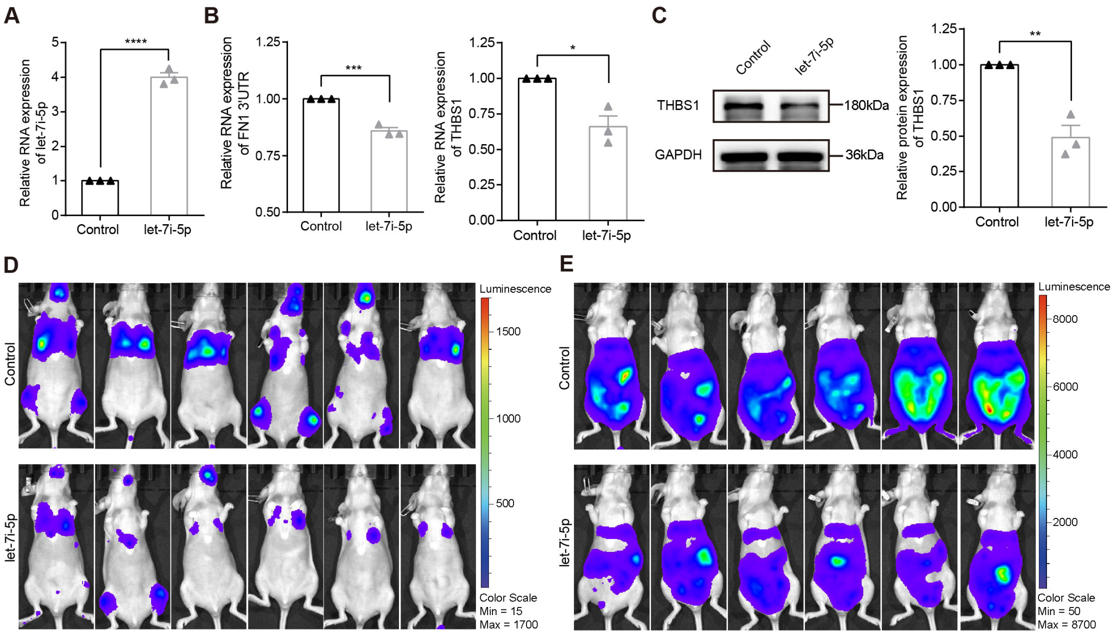

Figure S9. The transfection efficiency of stable-overexpressed let-7i-5p in HGC27 cells was validated by real-time PCR (A). The relative RNA levels of *FN1* 3′-untranslated region (UTR) and *THBS1* were determined by real-time PCR (B). The relative protein levels of THBS1 and corresponding analysis were determined by western blot and ImageJ software in HGC27 cells (C). Tumor volume was monitored using the in vivo optical imaging system for 9 weeks after tail vein injection (D). Tumor volume was monitored using the in vivo optical imaging system for 5 weeks after intraperitoneal injection (E). The data are presented as a histogram of the mean ± SEM of three independent experiments and compared by Student's *t*-test (*$P < 0.05$, ** $P < 0.01$, *** $P < 0.001$, **** $P < 0.0001$, n = 3).

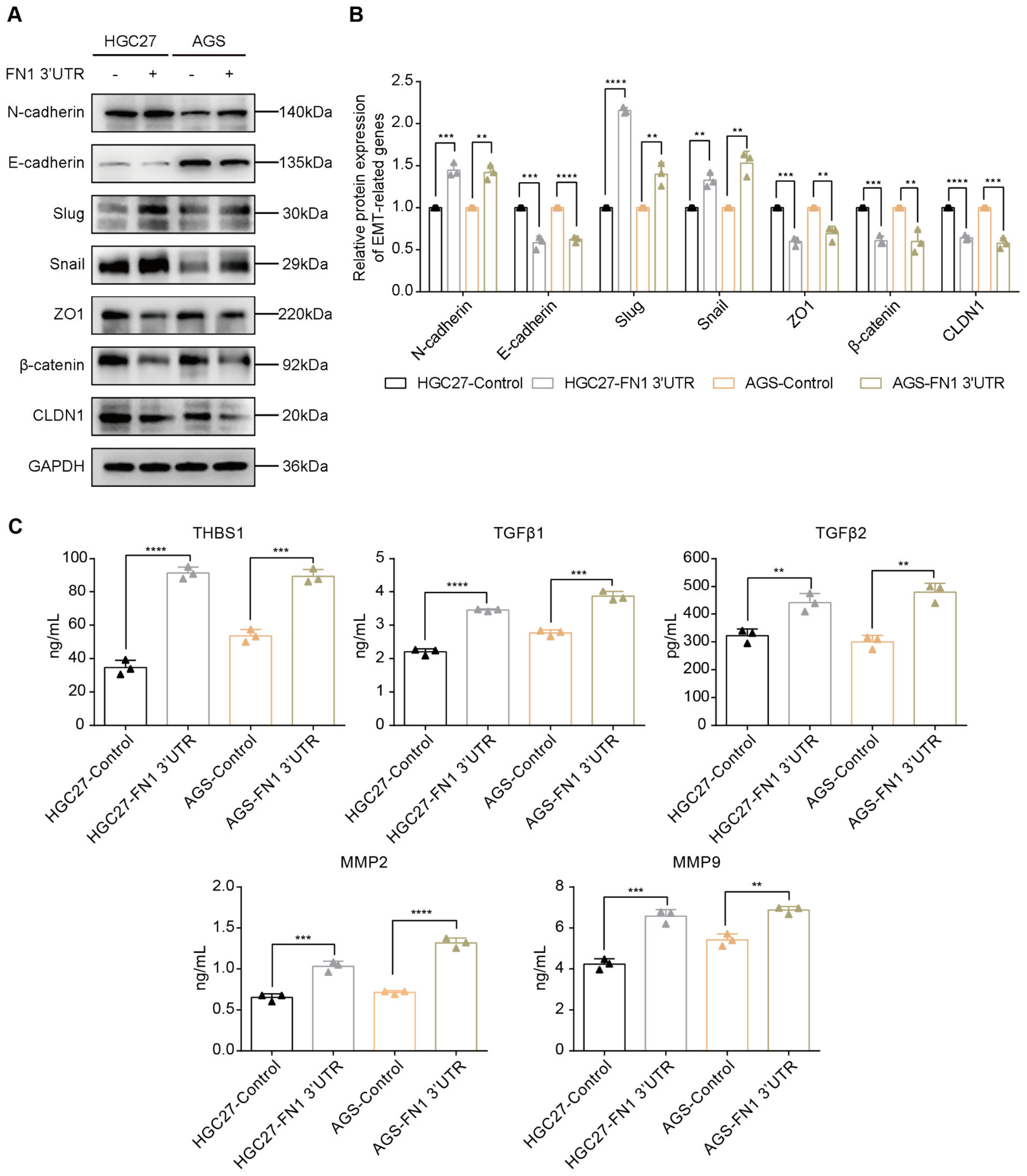

Figure S10. Western blot assays and corresponding analysis by ImageJ software showed the relative protein expressions of epithelial-mesenchymal transition markers in *FN1* 3′-untranslated region (UTR) overexpressed and negative control cells (A, B). Enzyme-linked immunosorbent assays evaluated the secretions of THBS1, TGFβ1, TGFβ2, MMP2, and MMP9 in *FN1* 3′-UTR overexpressed and negative control cells (C). The data are presented as a histogram of the mean ± SEM of three independent experiments in B and C and compared by Student’s *t*-test (**P* < 0.05, ** *P* < 0.01, *** *P* < 0.001, **** *P* < 0.0001, n = 3).

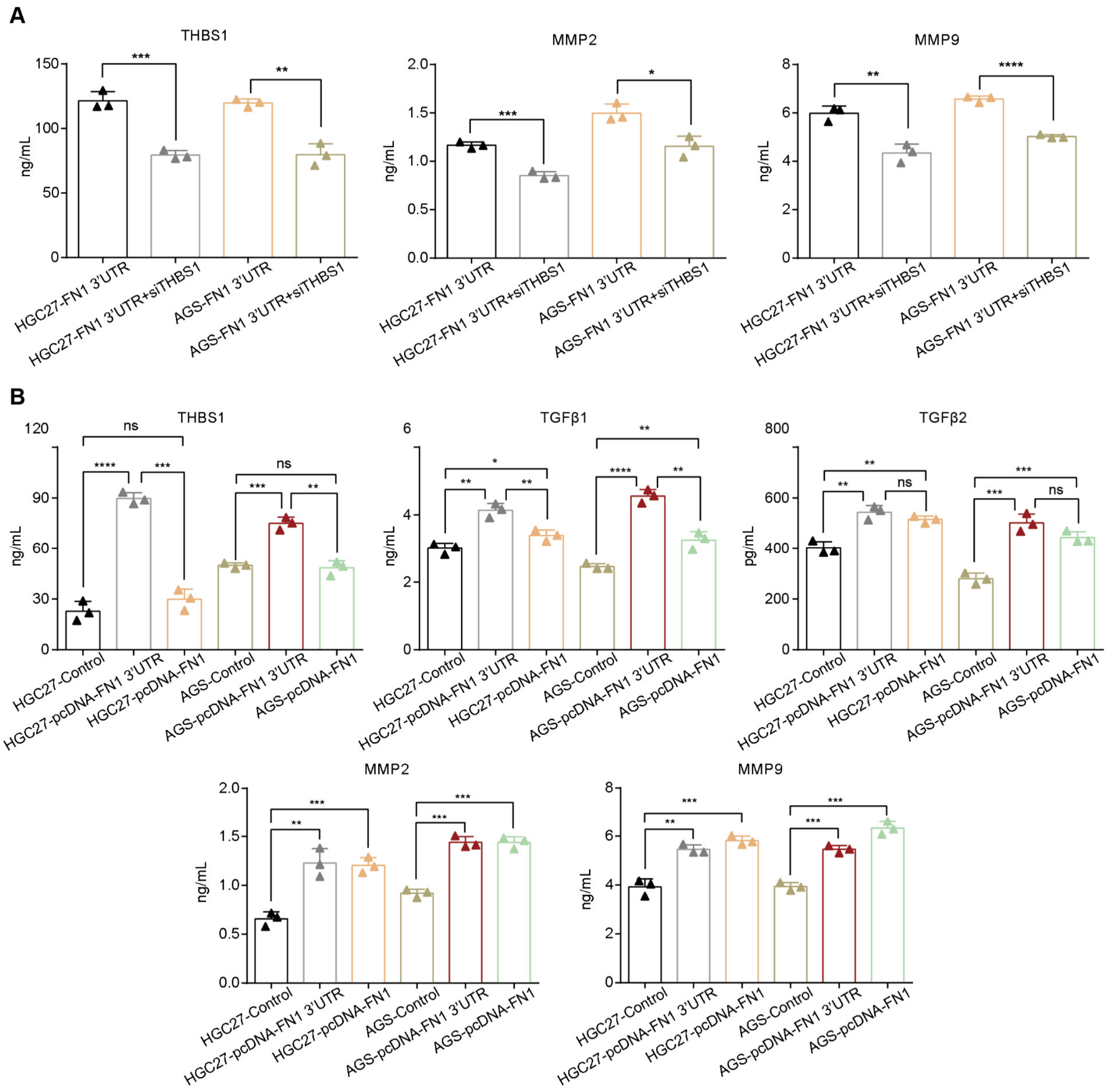

Figure S11. The alternations in secretions of THBS1, MMP2, and MMP9 after siTHBS1 transfection were determined by performing ELISA in *FN1* 3′-untranslated region (UTR) overexpressed cells (A). The secretions of THBS1, TGFβ1, TGFβ2, MMP2, and MMP9 were determined by performing ELISA using *FN1* 3′-UTR overexpressed, FN1 protein overexpressed, and negative control cells (B). The data are presented as a histogram of the mean ± SEM of three independent experiments and compared by Student's *t*-test (*$P < 0.05$, ** $P < 0.01$, *** $P < 0.001$, **** $P < 0.0001$, n = 3).

Table S1. Demographic and pathological characteristics of GC patients in the Chinese Medical University (CMU) cohort1 and cohort2.

able S2. RNA-seq data of HGC27-*FN1* 3′-untranslated region (UTR) and HGC27-Control cells.

Table S3. Differentially expressed genes (DEGs) selected from RNA-seq in HGC27-*FN1* 3′-untranslated region (UTR) and HGC27-Control cells.

Table S4-S6. miRNA-seq data and analyses of HGC27-*FN1* 3′-untranslated region (UTR) and AGS-*FN1* 3'UTR cells.

Table S7-S22. The intersect genes from both 16 miRNAs predicted target genes and the 241 up-regulated differentially expressed genes (DEGs) from RNA-seq.

able S23. 66 target genes screened with the help of expression profiles and prognostic information from the ACRG cohort.

Table S24. Oligonucleotides for qRT-PCR, miRNA and siRNA construction.
