# Supplementary material for: FN1 mRNA 3'-UTR supersedes traditional fibronectin 1 in facilitating the invasion and metastasis of gastric cancer through the FN1 3'-UTR-let-7i-5p-THBS1 axis
Source: Theranostics. 2023 Sep 25;13(14):5130–50. doi: 10.7150/thno.82492 (PMC10526670; doi:10.7150/thno.82492)
Supplement: Supplementary file 2 — Supplementary tables. [file thnov13p5130s2.zip › Supplementary Tables/Table S1.docx]

**Table S1.** Demographic and pathological characteristics of GC patients in the Chinese Medical University (CMU) cohort-1 and cohort-2.

| Characteristic | CMU cohort-1 (n = 222) | | | | | | CMU cohort-2 (n = 102) | | |
| --- | --- | --- | --- | --- | --- | --- | --- | --- | --- |
|  | FN1 3‘UTR expression | | *P* value | FN1 CDS expression | | *P* value | FN1 protein expression | | *P* value |
|  | High (n = 146) | Low (n = 76) |  | High (n = 115) | Low (n = 107) |  | High (n = 51) | Low (n = 51) |  |
| **Sex** |  |  | 0.192 |  |  | 0.904 |  |  | 0.221 |
| Male | 110 | 51 |  | 83 | 78 |  | 43 | 38 |  |
| Female | 36 | 25 |  | 32 | 29 |  | 8 | 13 |  |
| **Age** |  |  | 0.392 |  |  | 0.429 |  |  | 0.233 |
| ≤ 60 | 68 | 40 |  | 53 | 55 |  | 25 | 31 |  |
| > 60 | 78 | 36 |  | 62 | 52 |  | 26 | 20 |  |
| **Histology** |  |  | 0.136 |  |  | 0.128 |  |  | 0.830 |
| Differentiated | 73 | 30 |  | 58 | 45 |  | 16 | 15 |  |
| Undifferentiated | 73 | 46 |  | 57 | 62 |  | 35 | 36 |  |
| **Primary site** |  |  | 0.878 |  |  | 0.211 |  |  | 0.586 |
| Upper | 22 | 12 |  | 20 | 14 |  | 5 | 2 |  |
| Middle | 21 | 8 |  | 13 | 16 |  | 11 | 10 |  |
| Lower | 91 | 49 |  | 71 | 69 |  | 35 | 38 |  |
| Others | 12 | 7 |  | 11 | 8 |  | 0 | 1 |  |
| **Tumor size** |  |  | 0.094 |  |  | 0.463 |  |  | 0.843 |
| ≤ 5cm | 75 | 48 |  | 61 | 62 |  | 26 | 27 |  |
| > 5cm | 71 | 28 |  | 54 | 45 |  | 25 | 24 |  |
| **Bormann type** |  |  | 0.246 |  |  | 0.499 |  |  | 0.167 |
| EGC | 6 | 8 |  | 8 | 6 |  | 2 | 3 |  |
| I | 7 | 2 |  | 4 | 5 |  | 1 | 4 |  |
| II | 18 | 16 |  | 13 | 21 |  | 1 | 3 |  |
| III | 96 | 49 |  | 79 | 66 |  | 40 | 39 |  |
| IV | 13 | 7 |  | 11 | 9 |  | 7 | 2 |  |
| **Lauran’s Classification** |  |  | 0.673 |  |  | 0.627 |  |  | - |
| Intestinal | 61 | 34 |  | 51 | 44 |  | - | - |  |
| Diffuse | 85 | 42 |  | 64 | 63 |  | - | - |  |
| **pT stage** |  |  | **0.025^a^** |  |  | **0.042^a^** |  |  | **0.028^a^** |
| pT1 | 6 | 8 |  | 6 | 8 |  | 2 | 3 |  |
| pT2 | 34 | 19 |  | 21 | 32 |  | 7 | 3 |  |
| pT3 | 43 | 30 |  | 36 | 37 |  | 15 | 29 |  |
| pT4 | 63 | 19 |  | 52 | 30 |  | 27 | 16 |  |
| **pN stage** |  |  | **0.031^a^** |  |  | 0.707 |  |  | 0.534 |
| pN0 | 41 | 35 |  | 35 | 41 |  | 13 | 14 |  |
| pN1 | 20 | 14 |  | 20 | 14 |  | 10 | 6 |  |
| pN2 | 34 | 10 |  | 25 | 19 |  | 8 | 14 |  |
| pN3a | 33 | 11 |  | 23 | 21 |  | 9 | 9 |  |
| pN3b | 18 | 6 |  | 12 | 12 |  | 11 | 8 |  |
| **pM stage** |  |  | 0.438 |  |  | 0.652 |  |  | 0.647 |
| pM0 | 139 | 74 |  | 111 | 102 |  | 49 | 48 |  |
| pM1 | 7 | 2 |  | 4 | 5 |  | 2 | 3 |  |
| **LBVI** |  |  | 0.623 |  |  | 0.316 |  |  | - |
| Yes | 43 | 20 |  | 36 | 27 |  | - | - |  |
| No | 103 | 56 |  | 79 | 80 |  | - | - |  |

n, number of patients; EGC, early gastric cancer; LBVI, lymphatic and/or blood vessel invasion.

^a^[bold] means *P* value < 0.05, the difference was considered statistically significant.
